# Pain’s Adverse Impact on Training-Induced Performance and Neuroplasticity: A Systematic Review

**DOI:** 10.1007/s11682-021-00621-6

**Published:** 2022-03-18

**Authors:** Nikola Stanisic, Birgitta Häggman-Henrikson, Mohit Kothari, Yuri Martins Costa, Limor Avivi-Arber, Peter Svensson

**Affiliations:** 1 Department of Orofacial Pain and Jaw Function, Malmö University, Malmö, Sweden; 2 Scandinavian Center for Orofacial Neurosciences (SCON), Aarhus, Denmark; 3 Department of Clinical Medicine, Hammel Neurorehabilitation Center and University Research Clinic, Aarhus University, Aarhus, Denmark; 4 JSS Dental College and Hospital, JSS Academy of Higher Education and Research, Mysore, India; 5 Department of Biosciences, Piracicaba Dental School, University of Campinas, Sao Paulo, Brazil; 6 Prosthodontics and Oral Physiology, Faculty of Dentistry, University of Toronto, Toronto, ON Canada; 7 Section for Orofacial Pain and Jaw Function, Institute for Odontology and Oral Health, Aarhus University, Aarhus, Denmark

**Keywords:** Exercise, Neuronal plasticity, Nociception, Transcranial magnetic stimulation, Motor function

## Abstract

Motor training is a widely used therapy in many pain conditions. The brain’s capacity to undergo functional and structural changes i.e., neuroplasticity is fundamental to training-induced motor improvement and can be assessed by transcranial magnetic stimulation (TMS). The aim was to investigate the impact of pain on training-induced motor performance and neuroplasticity assessed by TMS. The review was carried out in accordance with the PRISMA-guidelines and a Prospero protocol (CRD42020168487). An electronic search in PubMed, Web of Science and Cochrane until December 13, 2019, identified studies focused on training-induced neuroplasticity in the presence of experimentally-induced pain, 'acute pain' or in a chronic pain condition, 'chronic pain'. Included studies were assessed by two authors for methodological quality using the TMS Quality checklist, and for risk of bias using the Newcastle–Ottawa Scale. The literature search identified 231 studies. After removal of 71 duplicates, 160 abstracts were screened, and 24 articles were reviewed in full text. Of these, 17 studies on acute pain (n = 7) or chronic pain (n = 10), including a total of 258 patients with different pain conditions and 248 healthy participants met the inclusion criteria. The most common types of motor training were different finger tasks (n = 6). Motor training was associated with motor cortex functional neuroplasticity and six of seven acute pain studies and five of ten chronic pain studies showed that, compared to controls, pain can impede such trainings-induced neuroplasticity. These findings may have implications for motor learning and performance and with putative impact on rehabilitative procedures such as physiotherapy.

**Supplementary Information:**

The online version contains supplementary material available at 10.1007/s11682-021-00621-6.

## Introduction

Neuroplasticity can be defined as the brain’s ability to reorganize or undergo functional and structural changes. It was long postulated that neuroplasticity was limited only to the critical period during brain development (Michelini & Stern, 2009). However, during the past decades, it has been widely recognized that neuroplasticity is the normal ongoing state of the human brain throughout the life span (Pascual-Leone et al., 2005). This feature of the brain is considered as one of the foundations for acquisition of new motor skills. In everyday life, we use different motor skills that we have acquired gradually through training and changes in our environment, e.g., walking, driving a car, riding a bicycle or chewing food (Chang, 2014; Lohse et al., 2014).

The neuroplastic changes associated with the training of a skill, i.e., motor training-induced neuroplasticity, are thought to play an important role in the performance of the skill being trained and can be reflected in measures such as accuracy, precision and speed. It has been suggested that training-induced neuroplasticity may be dependent on a number of factors, including the complexity of the skills being trained, training time, motivational conditions, age and the muscle groups activated during skill training to name a few (Duchateau et al., 2006; Hellmann et al., 2011; Wulf et al., 2010).

There are also reports that the presence of pain can have an impact on training-induced neuroplasticity and on the motor performance when executing a motor skill (Bank et al., 2013; Hodges & Tucker, 2011). The effect pain has on motor performance can both be subtle, e.g., redistribution of activity within/between muscles, increased variability, and more salient, e.g., avoidance of the motor behaviour causing or increasing the pain (Akhter et al., 2014; Hodges & Smeets, 2015; Hodges & Tucker, 2011).

Chronic pain conditions, as for example chronic lower back pain, which often result in limitations in motor performance, are common conditions and conventional treatments often involve different types of skill training to reduce disability and pain. Movements engaged during skill training will activate different corticomotor pathways in the brain to achieve appropriate, precise, and effective motor control and facilitate rehabilitation of the motor performance (Gurevich et al., 1994; Kosek et al., 2013). However, conventional rehabilitation and skill training programs may not optimally restore impaired motor performance in patients with chronic pain, compared to pain-free patients, due to a negative effect on training-induced neuroplasticity. Despite the importance of this topic in rehabilitation medicine, the effect of pain on training-induced neuroplasticity and the subsequential impact this has on motor skill acquisition and improvement, is not well understood.

Transcranial magnetic stimulation (TMS), a non-invasive brain imaging technique, can be used to assess training-induced neuroplasticity in corticomotor pathways following motor skill training (Rothwell, 2018). There is some evidence that both acute and chronic pain can affect the plasticity of the motor cortex—as assessed by TMS—following motor skill training, although reported findings are not conclusive. Thus, a reduction in corticomotor excitability has been reported for patients with both chronic and acute pain (Dettmers et al., 2001; Krause et al., 2006). In contrast, both chronic pain (e.g., phantom limb pain) (Dettmers et al., 2001) and acute pain have also been shown to increase motor cortex excitability under certain circumstances (Romaniello et al., 2000).

The aim of this systematic review was to investigate the impact of pain (acute or chronic) on training-induced motor performance and neuroplasticity as assessed by TMS.

## Materials and methods

### Protocol

This study followed a protocol that was registered in Prospero (CRD42020168487) and was carried out in accordance with the Preferred Reporting Items for Systematic Reviews and Meta-Analyses (PRISMA) Statement (Moher et al., 2009).

### Inclusion and exclusion criteria

Eligibility criteria were formulated using PICO (population, intervention, comparison and outcome) to identify clinical studies published in English and focused on pain and training-induced neuroplasticity assessed with TMS in humans.

- **Populations**: Healthy humans with experimentally induced acute pain or humans with a chronic pain condition.
- **Intervention:** Short-term motor task training in the presence of pain (acute or chronic).
- **Comparison**: Healthy humans with no pain.
- **Primary outcome**: Neuroplasticity assessed with TMS targeting motor cortex areas of the corresponding muscles involved in the training task performed in the presence of pain.
- **Secondary outcome:** Behavioral and functional outcomes.

Articles were excluded if a) the population had psychiatric or neurological disorders, b) participants did not perform an active form of training, c) participants performed long term training (e.g., sport athletes) and d) the training was combined with other interventions (e.g., paired associative stimulation).

### Literature search

An electronic search was carried out in PubMed, Cochrane and Web of Science until December 13, 2019. The search strategy was developed for PubMed with a combination of MeSH terms and free text terms in cooperation with an experienced research librarian (Martina Vall) and then adapted to both Cochrane and Web of Science. The search was designed to identify studies that assessed the possible effect of acute or chronic pain on training-induced neuroplasticity assessed with TMS. Table 1 provides the full search strategy for PubMed. There was no limitation on study design or language in the search. The electronic search was combined with a hand search of the reference lists of the included articles to identify additional studies. Grey literature, letters to the editor and editorials were not included, and authors were not contacted for additional information.

**Table 1:** PubMed Search strategy

Search	Search String
1. Plasticity	("Sensorimotor Cortex"[Mesh] OR corticomotor plasticity[tiab] OR corticomotor control[tiab] OR corticomotor pathway*[tiab] OR sensorimotor cortex[tiab] OR neuroplasticity[tiab] OR "Neuronal Plasticity"[Mesh] OR cortical plasticity[tiab] OR cortical neuroplasticity[tiab] OR brain plasticity[tiab] OR Neuronal Plasticity[tiab])
2. TMS	(TMS[tiab] OR Transcranial Magnetic Stimulation[tiab] OR "Transcranial Magnetic Stimulation"[Mesh])
3. Exercise	(exercise[Mesh] OR rehabilitation[Mesh] OR rehabilitat*[tiab] OR exercis*[tiab] OR train*[tiab] OR physical therapy modalities[Mesh] OR physical therapists[Mesh] OR physiotherap*[tiab] OR physical therapy specialty[Mesh] OR kinesio*[tiab]OR learning[tiab] OR (physical[tiab] AND therap*[tiab]))
4. Pain	Pain OR nociception
5. Result	#1 AND #2 AND #3 AND #4

### Article selection

Two authors (BHH, NS) independently screened all titles and abstracts for potential eligibility. If at least one author deemed an article to be of potential interest, it was retained for the full text assessment. The full text assessment was carried out independently by the same authors to determine if articles met the inclusion criteria. Any disagreements were resolved by discussion between the investigators and if needed by a third author (MK).

### Data extraction

Data extraction was carried out by two authors (NS, MK) and checked by a third author (BHH). The data extracted from the individual studies were: first author, publication year, aim, study population, study setting, study design, methods, outcomes, and study summary. All data supporting the findings of this study are available within the article and its 19. A record of the database searches is available on Prospero (CRD42020168487). If results allowed, primarily with regard to reported TMS outcome measures for neuroplasticity and elapsed time between training and assessment, a meta-analysis was planned using a random effects model and using the I^2^ statistic for assessing heterogeneity among studies.

### Quality assessment

Assessment of risk of bias in the included studies was carried out by two authors (NS, LAA) using the Newcastle–Ottawa Scale (NOS) for case–control studies (Stang, 2010). In addition, the included studies were assessed by two authors (NS, YC) with the TMS Quality checklist (Chipchase et al., 2012). For both of these instruments, any disagreement between the authors were resolved by discussion, and if needed by a third author (BHH). The NOS evaluates the risk of bias by looking at eight items categorized in three different domains: selection, comparability and exposure. NOS uses a star system where the studies with the highest quality are awarded a maximum of one star for each item with exception for the item related to comparability where the assignment of two stars are allowed providing a total score between 0 and 9. The TMS Quality checklist includes eight items on participant characteristics, 20 items on TMS methodology, of which three items specifically concern paired pulse only, and two items on analysis. In total, the checklist includes 26 factors that can be reported and/or controlled for in studies using single pulse TMS, and 29 factors that can be reported and/or controlled for in studies using paired pulse TMS. The checklist items were assessed as: “Yes”, “No”, or “Not applicable”, and the number of these respective answers was reported together with the total percentage of “Yes” answers with the number of attainable criteria as denominator.

## Results

### Literature search

**Fig. 1:**
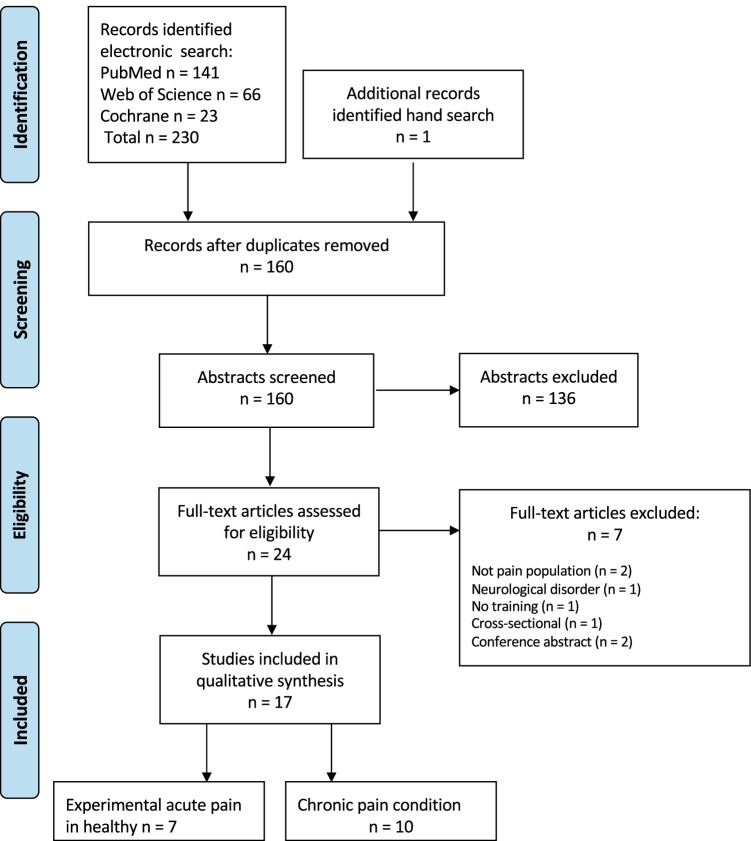
PRISMA flow chart showing numbers of included and excluded studies

The electronic literature search in PubMed, Cochrane and Web of Science, identified a total of 231 articles (Fig. 1). After removal of 71 duplicates, 160 unique abstracts were screened, and 24 articles were reviewed in full text. Of the 24 articles, 7 articles were excluded (Bradnam et al., 2016; Daligadu et al., 2013; Massé‐Alarie et al., 2015; Massé-Alarie et al., 2017a; McCambridge et al., 2018; Rittig-Rasmussen et al., 2013; Volz et al., 2012) (Table 2) and 17 articles published between 2007 and 2018 met the inclusion criteria, i.e., reported the impact of pain on training-induced neuroplasticity assessed by TMS (Table 3). The electronic search was complemented with a hand search of the reference lists of included articles.

**Table 2:** Articles excluded from the study during full-text assessment and the main reasons for exclusion (n = 7)

Main reason for exclusion	First author Year
No pain population	Rittig-Rasmussen 2013, Volz 2012
Neurological disorder	Bradnam 2016
No training performed	Massé-Alarie 2017
Cross-sectional data	Daligadu 2013
Conference abstract	Massé-Alarie 2015, McCambridge 2018

**Table 3:** Data extracted from the included papers (n = 17)

**First author** **Year**	**Aim**	**Study population** Number Female/Male Age: Mean (SD) Study setting Study design	**Methods**	**Results** Primary outcomes: TMS Secondary outcomes: Functional	**Study summary** related to training and pain	**Comments**
Baarbe 2018	To determine if cerebellar inhibition would reduce to the same extent in patients with mild recurrent neck pain as in healthy individuals following the same MA task	Neck pain n: 27 F/M: 16/11 Age: 21.1 (1.9) yrs Healthy controls n: 12 F/M: 2/10 Age: 22.4 (2.2) yrs *Study setting:* University laboratory *Study design:* Experimental study	**Experimental protocol** 1) Short test block (baseline) 2) TMS (baseline) 3) Manipulation:—Neck pain *spinal manipulation (SM) (n* = *14) sham manipulation (n* = *13)* *- Healthy no intervention (n* = *12)* 4) Task training 5) TMS (post-training) **Task** Typing 8-letter sequence of 4 letters as quickly and accurately as possible *Short test block 1–2 min, MA 10 min, short test block 1–2 min. Total training time: 15 min* **Outcome measures** **TMS** Single pulse left FDI M1 (target muscle): Stimulator output adjusted to elicit test MEP ≈ 0.5 mV in peak-to-peak amplitude. rMT and test MEP before + after manipulation/sham and typing task **Functional** Response time and accuracy in test block before and after MA	**TMS:** MEP amplitude before 0.67 ± 0.12 mV and after intervention: 0.64 ± 0.16 mV. MEP amplitudes: no interaction effects of group vs. time (p = 0.3) or effects of time (p = 0.3). rMT: no effects of group vs. time (p = 0.7) of effects of time (p = 0.6) **Functional:** Neck pain + SM: reduced response time, marginal increase accuracy Neck pain + sham: moderate reduction response time, improved accuracy Healthy: marginally reduced response time, no change accuracy	MEP amplitudes and rMT did not change in any group pre- to post-intervention. The stimulator output for the test MEP was also the same pre- and post-motor training	Neck pain group had subclinical recurrent neck pain
Boudreau 2007	To determine if i) short-term novel tongue-protrusion training in humans is associated with rapid neuroplasticity of the tongue MI ii) intra-oral tonic pain affects the tongue MI neuroplasticity and tongue-protrusion training performance	Healthy individuals n:9 F/M: 2/7 Age: 24 (1.1) yrs *Study setting:* University laboratory *Study design:* Experimental study	**Experimental protocol** 1) TMS (baseline) 2) Experimental pain (capsaicin/ inert lotion) 3) Task training 4) TMS (post-training) **Experimental pain** Application area: tongue musculature Capsaicin (cream) (n = 9) Placebo (inert lotion) (n = 9) **Task** TPT: maintain cursor within moving target box displayed on computer screen. *Total training time: 15 min* **Outcome measures** **TMS** Single pulse tongue M1 (target muscle) and FDI M1 (control muscle): MT determined for relaxed muscles and defined as lowest % TMS output that produced 5 out of 10 discernable MEP for each muscle (i.e., ≥ 10uV for the tongue and ≥ 50uV for the FDI). TMS-MEP stimulus–response curves constructed in increments of 10% threshold up to a maximum of 85% TMS output. 10 stimuli delivered at each TMS increment, ISI 8-10 s. M1 excitability defined as relationship between % TMS output required to elicit an MEP in both muscles. TMS-MEPs were rectified and evaluated in terms of the AUC. TMS-MEP stimulus–response curves before and immediately after each task training session **Functional** Performance score (%) for maintaining cursor within target box in TPT	**TMS-MEP S-R curve:** Capsaicin: NS difference (p = 0.311) Placebo: significant enhanced MEPs at 1.4 and 1.5 TMS intensity (p = 0.007 and p = 0.005, respectively) **rMT:** Capsaicin: 46.7 ± 3.3% vs 46.6 ± 3.7% (p = 0.871) Placebo: decrease 48.3 ± 4.1% vs 45.1 ± 3.4% (p < 0.001) NS difference pre-training TMS-MEP stimulus response curves vehicle vs capsaicin (p = 0.501) **Functional:** Significant increase with time for placebo (p < 0.0001) and capsaicin (p < 0.012). Compared to placebo, capsaicin had significantly lower performance score with less improvement **Additional outcomes:** Mean performance score for capsaicin TPT session not significantly correlated to AUC pain intensity (p = 0.91)	Short-term TPT is associated with rapid neuroplasticity of tongue MI and significant increases in TPT performance, but the presence of experimental pain interferes with these neuroplastic and performance changes The study also showed that the perceived pain intensity was not correlated to the overall mean performance scores for the capsaicin TPT session	
Dancey 2019	To determine the interactive effect of acute tonic pain and early motor learning on corticospinal excitability as measured by TMS	Healthy individuals n:24 F/M: 18/6 Age: 20.2 (1.3) yrs *Study setting:* University laboratory *Study design:* Experimental study	**Experimental protocol** 1) TMS, NPRS (baseline) 2) Experimental pain (capsaicin/ Inert lotion) 3) TMS, NPRS (post application) 4) Task training 5) TMS, NPRS (post-training) 6) Task training (24-48 h from baseline (w/o capsaicin) *Retention* **Experimental pain** Application area: Lateral aspect dominant elbow Capsaicin (cream) (n = 12) Placebo (inert lotion) (n = 12) **Task** Tracing sequences sinusoidal waves (varying amplitude and frequency) on touchpad with dominant thumb*. Training: pre-test 4 min, post-test 4 min, retention 4 min, MA 15 min. Total training time: 27 min* **Outcome measures** **TMS** Single pulse dominant APB M1 (target muscle): rMT utilizing lowest stimulatory intensity that in 5 of 10 sessions evoked MEP of at least 0.05 mV, while muscle was at rest. TMS-IO curve intensity established using each participants rMT to stimulate 90–140% of rMT in 10% increments. 12 stimuli at each stimulus intensity with ISI 5 s (72 stimuli / IO curve session) TMS IO curves at baseline, post-application, and following MA **Functional** Mean error (%) on motor task	**TMS:** No effect of time on IO slopes following capsaicin or placebo, or post MA Significant increase in IO slope after motor acquisition for control group. NS change for capsaicin group **Functional:** Relative to the pre-test: both groups decreased error post-MA and following retention (all p < 0.001) Placebo: 48.7% decrease in mean error post MA, further 21.9% decrease at retention Capsaicin: 35.2% decrease in mean motor error following MA and a subsequent 10.7% decrease at retention	The acute tonic pain in this study was shown to negate the increase in IO slope observed for the control group despite the fact that motor performance improved similarly to the control group following acquisition and retention Clearly there is a link between motor and sensory systems and the effects of pain on motor learning may be due to cortico-thalamic, cortico-cerebellar, or cortico–cortico loops	
De Martino 2018a	To investigate if i) combined injection of NGF and DOMS provokes greater muscle soreness and disability, increased hyperalgesia, and reduction of maximal grip force compared with intramuscular injections of NGF alone, ii) the sensorimotor cortical neuroplastic consequences of muscle soreness induced by DOMS in a muscle pre-sensitized by intramuscular injection of NGF	Healthy individuals *NGF* n:12 F/M: 7/5 Age: 25.1 (1.6) yrs *NGF* + *DOMS* n:12 F/M: 7/5 Age: 26.7 (1.2) yrs *Study setting:* University laboratory *Study design:* Experimental study	**Experimental protocol** 4 experimental sessions over 6 days. Day-0, 2, 4 and 6: 1) Pain related questionnaires 2) TMS (Not Day 2) 3) Sensory and motor assessment: grip force, wrist extensor force, and PPTs **Experimental pain** Both groups: NGF injection right ECRB muscle at end of session Day-0, 2 + 4 NGF + DOMS group Day-4: eccentric exercise before receiving NGF injection to provoke DOMS **Task** Right hand eccentric contractions from maximally extended wrist position to maximally flexed wrist position, duration of at least 4 s while holding a weight (max 25 kg). Sets of 5 repetitions. First set, 90% of MVC repeated until participant was not able to control the eccentric contraction over 4 s. Load then progressively reduced in steps of 10% MVC ending at load of 50% MVC in final set **Outcome measures** **TMS** Single pulse ECRB M1 (target muscle): rMT threshold defined as intensity at which 5 of 10 stimuli evoked MEP with amplitude of at least 50 uV while muscle was in rest. Stimulus intensity set at 120% rMT. Motor cortical map representing the ECRB activation recorded based on MEPs evoked every 6 s with a total of 5 stimuli at each site on the stimulation grid. All grid sites randomly stimulated from the hotspot until no MEP was recorded (defined as < 50uV) in all five stimuli at all border sites. If average peak-to-peak amplitude of 5 MEPs evoked at site was greater than 50 uV, site was considered active. Map volume calculated as sum of MEPs from active sites. CoG defined as the amplitude-weighted center of the map	**TMS:** Map volume: Progressive increase Day-0 to 4 in both groups (p < 0.01). Day-6, NGF group increase compared with Day-0, (p < 0.01), NGF + DOMS group reduction compared with Day-4 (p = 0.01) MEP: Compared with Day-0, number of active sites and MEP amplitudes on hot spot increased Day-4 (p < 0.01) and Day-6 (p < 0.02) No difference in rMT (p = 0.5), longitude or latitude center of gravity (CoG) (p = 0.55) and p = 0.07, respectively) between groups and days	NGF induced soreness extended the ECRB motor map which subsequently was depressed by DOMS	
De Martino 2018b	To assess changes in sensorimotor cortical excitability during experimental muscle soreness across several days provoked by eccentric exercise of wrist extensors	Healthy individuals n:12 F/M: 6/6 Age: 24 (3.2) yrs *Study setting:* University laboratory *Study design:* Experimental study	**Experimental protocol** 5 experimental sessions over 6 days. Training session (day before baseline) not analyzed Day 0 (baseline): 1) Pain related questionnaires and TMS 2) Max grip force and max wrist extension force, PPT as baseline 3) Eccentric exercise right wrist extensor muscles to induce DOMS 4) 2 h after eccentric exercise: postexercise session on the same day and TMS measures performed after Day 2 and 6: Data collection repeated **Experimental pain and task** See De Martino 2018a (same task but no NGF injections) **Outcomes measures** **TMS** See De Martino 2018a	**TMS:** Number of active sites and map volume significantly reduced at 2-h and day 2 compared with baseline (p < 0.001 and p < 0.0026, respectively). The map area still reduced at Day 6 compared with Baseline (p = 0.026) NS effects for: rMT (p = 0.16), MEPs (p = 0.3), number of discrete peaks (p = 0.33), and position of CoG (long p = 0.41 lat p = 0.27)	DOMS is associated with decreased number of MEP active sites and motor map area of the affected muscle	
Hoeger Bement 2014	To assess corticomotor excitability in fibromyalgia during a noxious stimulus before and after fatiguing exercise and examine associations with pain perception	Fibromyalgia n:15 F/M: 15/0 Age: 53.7 (9.9) yrs *Study setting:* University laboratory *Study design:* Experimental study	**Experimental protocol** 3 sessions: 1 familiarization, and 2 experimental Familiarization session: subjects introduced to pressure pain device and TMS Randomized experimental sessions (S1, S2) NRS and TMS before and after: (S1) 30 min rest or (S2) Task training **Experimental pain** Noxious stimuli with pressure pain device on right index finger **Task** Submaximal isometric contraction left elbow flexor muscles until task failure or patient required to stop **Outcome measures** **TMS** Single pulse left brachioradialis muscle M1 (target muscle): MEP determined by stimulating the motor cortex at intensity 40–70% of MSO that produced MEP in the muscle at least 3 of 6 trials. Stimulation intensity 120% of the MEP. TMS delivered in sets of 4 pulses (3 s apart) at 6 separate time points before and after task training or rest. TMS before and after rest/contraction at 6 time points: before, start of pain test, midpoint of pain test, before end of pain test, immediately after pain test and 30 s after pain test	**TMS:** MEP amplitude: No changes during pain test in rest or task sessions (p = 0.45) After training, significant decrease during and immediately after pain test compared with baseline: 0 s (p = 0.03), 50 s (p = 0.006), 110 s (p = 0.01) and immediately after pain (p = 0.05) No differences for other time points or for rest sessions	Decrease in mean MEP amplitude but an interaction between pain response after exercise and pain induced changes in MEPs	
Ingham 2011	To investigate if pain interferes with plasticity, affecting acceleration of finger movement when the training task is painful, despite control of training task performance	*Study population:* Healthy individuals n: 9 F/M: 6/3 Age: 21.4 (2.3) yrs *Study setting:* University laboratory *Study design:* Experimental study	**Experimental protocol** 3 separate experimental sessions: 1) Injection of saline 2) TMS (baseline) 3) Task training (block 1–3) 1 block: 8 sets × 50 s, 10 s rest between sets for NRS 4) TMS after each task training block 5) TMS 5, 10 and 15-min post-training **Experimental pain model** Local pain: HS injection in trained FDI Remote pain: HS injection in infrapatellar fat pad on knee Control: IS injection FDI **Task** Brisk movements right index finger in opposite direction to TMS evoked movement. Each training set 50 s, followed by 10 s rest to minimize risk for fatigue. *Total training time: 8 min including 80 s rest* **Outcome measures** **TMS** Single pulse right FDI M1 (target muscle) and finger extensor M1 (control muscle): Stimulation intensity required to elicit MEPs in FDI with observed finger movement towards the thumb in 5 consecutive stimuli determined. Stimulation intensity set at 120% of this value, as this intensity provides consistent MEPs in the test muscle. 10 stimulations administered at baseline and after each of the 3 training sessions. Three additional blocks of stimuli delivered after training (5, 10 and 15 min). Peak amplitude of TMS-induced finger acceleration compared between conditions and between times **Functional** Number of finger movements and peak acceleration	**TMS:** TMS evoked peak acceleration after training was reduced in FDI pain and control following 3^rd^ training and 1^st^ recovery session (both p < 0.001) with no difference between FDI pain and control (p > 0.189) No difference in latency to onset of MEP amplitude between conditions (p = 0.94) or following training (p = 0.80) No difference MEP amplitude between conditions (p = 0.94) or following training (p = 0.16) **Functional:** Number of finger movements similar between sessions (p = 0.1). Peak acceleration increased during training and improvement rate was similar in all conditions (p = 0.65)	There was no change in FDI MEPs in any conditions. These data do not support direct effects of pain on training-induced plasticity of corticomotor pathways Remote pain may compromise learning due to distraction from the training task or other complex central pain processes	Small group (n = 9)
Massé-Alaire 2017b	To determine whether combining RPMS and motor training of the superficial MF better improved the corticomotor control of spine than training alone in chronic low back pain	*Study population:* Back Pain n:21 F/M: 10/11 Allocated to: *RPMS* Age: 33.2 (10.8) yrs n = 11 (2 dropouts) *Sham* Age: 42.1 (17.2) yrs n = 10 (1 dropout) *Study setting:* Neurostimulation laboratory *Study design:* Experimental study	**Experimental protocol** Task performed twice a day at home and 3 lab sessions (S1, S2, S3) over 1 week with intervention (RPMS) or sham before performing task supervised **S1 and S3:** 1) TMS, APA, muscle activation, VAS (baseline) 2) Task training and *RPMS (n* = *11) or Sham (n* = *10)* 3) TMS, APA, muscle activation, VAS (post-training) **S2:** 1) Task training and *RPMS (n* = *11) or Sham (n* = *10)* **Task** Isometric contraction lumbar MF (attention contraction deep MF, minimal activation of superficial MF and adjacent erector spinae) **Outcome measures** **TMS** Single pulse MF M1 (target muscle): AMT defined as TMS intensity eliciting at least 5 measurable MEP in the pre-activated MF, out of 10 trials Paired pulse MF M1 (target muscle): SICI probed by conditioning TMS (70% AMT) and test TMS at 120%. AMT; two ISI were tested with the conditioning TMS delivered 2 ms and then 3 ms before the test. SICF probed by conditioning TMS (90% AMT) delivered 1 ms after a test TMS at 100% AMT. In each paradigm, 8–10 unconditioned (test) MEP and 8–10 conditioned MEP were elicited For each participant, amplitudes of test MEP matched between pre-intervention at S1 and other time points (adjustment test TMS intensity) to ensure valid comparisons of conditioned MEP amplitudes **Functional** Onset of activation for each muscle and onset of APAs	**TMS:** Sham: No effects were detected in TMS outcomes **Functional:** Sham: Earlier APA at S3 compared to S1 (0.008)	Task training in the presence of chronic low back pain had no effect on any TMS measure	Small group (n = 9) with chronic low back pain Only results from the sham group eligible in the present review
Massé-Alaire 2016	To compare the effects of isometric activation (ISOM) of deep multifidi muscles (MF) and global activation of paravertebral muscles (GLOB) on MF postural activation and M1 function in a chronic low back pain population	*Study population:* Back Pain n:22 F/M: 8/ 14 Allocated to: *GLOB* Age 45.4 (18.1) yrs n = 11 (1 dropout) *ISOM* Age: 35.1 (11.4) yrs n = 11 *Study setting:* Neurostimulation laboratory *Study design:* Experimental study	**Experimental protocol** Task performed twice a day at home and 4 experimental sessions (S1-4) over 3 weeks (W1-W3) **S1 (W1):** 1) TMS, APA, VAS (baseline S1) 2) Supervised task training: ISOM/GLOB **S2-S3 (W2):** 1) Supervised task training: ISOM/GLOB **S4 (W3):** 1) TMS (baseline S4) 2) Supervised task training: ISOM/GLOB 3) TMS, APA, VAS (post task) **Task** GLOB: global activation of paravertebral muscles (hip extension); ISOM: Isometric contraction lumbar MF (attention contraction deep MF, minimal activation of superficial MF and adjacent erector spinae) **Outcome measures** **TMS** AMT, SICI, SICF (same as Massé-Alaire 2014) Paired pulse MF M1 (target muscle): LICI: conditioning TMS 120% AMT; 100 ms later test TMS 120% AMT. LICF: conditioning TMS (80% AMT); 15 ms later test MEP 120% AMT. In each paradigm, 8–10 unconditioned (test) MEP and 8–10 conditioned MEP were elicited For each participant, amplitudes of test MEP were matched between pre-intervention at first session (S1) and other time points (adjustment of test TMS intensity) to ensure valid comparisons of conditioned MEP amplitudes **Functional** Two rapid limb movements: bilateral shoulder flexion and unilateral hip extensions to study APA latency of MF, TrA/IO and EO muscles. Onset of activation determined by visual inspection and onset of APAs	**TMS:** AMT significant decrease for post-S4 (48.6 ± 11.7% MSO) vs. pre S4 (51.1 ± 11.4% MSO). No p-values reported, no between-group difference Ln-transform MEP amplitude, based on analysis n = 10 GLOB and n = 9 ISOM due to TMS artefacts: ISOM: significantly decreased S1 vs post S4 (p = 0.006) and pre-S4 vs post-S4 (p = 0.015). GLOB: no change (p > 0.05) Normalized SP duration: based on analysis n = 7 GLOB (not presented) and n = 9 ISOM due to EMG problems: ISOM: significant effect of time (p = 0.03), but not in pairwise comparisons between individual time points (S1 vs. pre-S4: p = 0.08; pre- vs. post-S4: p = 0.14) **Functional:** Bilateral shoulder flexion task: ISOM: significant effect of Time (p = 0.006); earlier MF-S onset at post-S4 than at S1 (p = 0.02), not changed in GLOB: (p = 0.73) Prone hip extension task: Group vs. Time interaction for MF onset. GLOB: NS difference ISOM: main effect of Time (p = 0.04) with MF-S onset earlier at post-S4 than at S1	Exercise decreased AMT in back pain patients. Isometric exercise modulated cortical inhibition and corticospinal excitability with decrease in MEP amplitude	Small groups analysed (n = 7 to n = 10)
Mavromatis 2017	To assess the effect of pain on changes in motor performance and corticospinal excitability during training of a novel motor task	*Study population:* Healthy population Allocated to: *Capsaicin* n:15 F/M: 6/9 Age: 26 (6) yrs *Control* n:15 F/M: 9/6 Age:27 (6) yrs *Study setting:* University laboratory *Study design:* Experimental study	**Experimental protocol** 1) TMS (baseline 1) 2) Experimental pain (Capsaicin/ placebo cream) 3) TMS (baseline 2) 4) Task training (10 blocks) *5)* TMS assessed after each training block; SICI assessed at halfway point and at end of training **Experimental pain** Application area: lateral border of the first metacarpal Capsaicin (cream) (n = 15) Placebo (inert lotion) (n = 15) **Task** Pinching force transducer between right thumb and index finger. Single session of 10 training blocks, measures early (1–2), mid (5–6) and late (9–10) training block. Each block with 15 continuous sequences **Outcome measures** **TMS** Single pulse FDI M1(target muscle): Intensity set to evoke average MEP of approximatively 1 mV with muscle fully relaxed (mean of 15 MEPs). AMT assessed with contraction of 5% of MVC, defined as the intensity that produced at least 5/10 MEPs with amplitude greater than 10% of the mean background EMG Paired pulse TMS for right FDI M1 (target muscle): SICI assessed with conditioned stimulus at 90% of AMT, intensity similar to the one used for single pulse measurements and kept throughout the entire experiment Task performed after second baseline measurements and after each training block, 15 single-pulse MEPs were recorded. SICI recorded halfway (block 5) and at end of the motor learning (block 10) **Functional** Movement time, accuracy and skill measure	**TMS:** MEP amplitudes Placebo: Increase between early- and mid-training periods (p = 0.005), followed by return to baseline in late training period (p = 0.995) Capsaicin: No increase from early- to mid-training (p = 0.713). No significant differences between any time periods SICI over time during motor training revealed NS effect of group (p = 0.319), time (p = 0.717) or group x time interaction (p = 0.983) F**unctional:** Both groups performed the task faster with training (p < 0.001), showed a significant improvement over time (p < 0.001), and with similar rate of improvement (p = 0.573) Subjects in the capsaicin group performed better throughout the training period (p = 0.029)	Pain did not negatively impact the acquisition of a novel motor task, however, it did have a negative effect on the training-related increase in corticospinal excitability observed in the control group	
Mendonca 2016	To determine the clinical and neurophysiological effects of the combination of tDCS and AE on a treadmill over 1 month to generate results of a new intervention and to understand how modulation of the M1 circuit leads to pain control	*Study population:* Fibromyalgia n: 45 F/M: 44/1 Age: 47.8 (12.1) yrs Allocated to: *tDCS* + *AE* n:15 *AE* n:15 *tDSC* n:15 *Study setting:* University laboratory *Study design:* Clinical, randomized, double-blind study	**Experimental protocol** 4-week task period: 5 days of training week 1, and 3 days of training week 2–4 1) NRS (pain and anxiety levels), PPT, QOL, mood, TMS (baseline 1 week before training task) 2) Task training 3) All variables after the fifth day of intervention 4) Task training 5) After task (T2) 6) Follow ups (1-month + 2-months post-training) **Task** AE treadmill: Intensity 60% of max heartrate week 1 and if possible 70% of max heartrate week 2 AE sham treadmill: 5% of resting heartrate maintained at minimum speed *Total training time: 30 min per session* tDCS + AE: active intervention of aerobic exercise training + tDCS AE: active intervention of aerobic exercise + placebo tDCS tDCS: placebo AE + active intervention for tDCS **Outcome measures** **TMS** Single pulse right adductor muscle of the thumb M1 (target muscle): MT lowest intensity for TMS pulse to generate peripheral response of at least 50 mV of amplitude. Same method used to determine MEP at 120% of the intensity found for the MT. 10 MEPs measured at each stage Paired pulse right adductor muscle of the thumb M1 (target muscle): ICF conditional pulse with intensity of 80% of the MT and test pulse with the MEP intensity. ISI 10 ms. ICI: same parameters but 2 ms ISI. 15 measures of ICF and ICI each, randomized between inhibition, facilitation and ME, totaling 45 pulses	**TMS:** NS effects found for any TMS outcomes for any group	Task training in the presence of fibromyalgia had no effect on any TMS measure	Only results from AE group eligible in the present review
Parker 2017	To i) provide a thorough analysis of corticomotor and intracortical excitability in people with chronic arthritic hand pain ii) examine the relationship between these measures and performance on a motor skill learning task	*Study population:* Arthritic hand pain n: 23 F/M: 17/5 Age: 72 (6) yrs Healthy controls n: 20 F/M: 14/6 Age: 71 (7) yrs *Study setting:* University laboratory *Study design:* Experimental study	**Experimental protocol** 1)TMS (baseline) 2)Task training 3) TMS (0- and 10-min post-training) **Task** 30-min voluntary finger twitches with auditory cue to target direction (opposite direction to baseline TMS induced finger twitches). *Total training time: 30 min* **Outcome measures** **TMS** Single pulse FDI M1 (targeted muscles): rMT defined as minimum stimulus intensity that elicited MEP with peak-to-peak amplitude of at least 50uV in a minimum of 4 out of a train of 8 stimuli, established by increasing stimulus intensity in 5% increments until MEPs were elicited, and then adjusted intensity in 1% intervals until rMT was determined. S-R-curve obtained by delivering 80 stimuli at 10% increments from 90 to 160% rMT, with 10 stimuli at each intensity Paired pulse TMS FDI M1 (target muscle): A block of 60 test stimuli delivered over the hot spot. 10 stimuli delivered for each condition. Two SICI assessed at 70% and 80% rMT and the test stimulus was 1 mV with an ISI of 2 ms. Two SICF assessed with conditioning stimuli being 1 mV and test stimulus 90% rMT with an ISI of 1.4 ms and 2.8 ms. LICI assessed with conditioning stimuli being 120% rMT and test stimulus was 1 mV with ISI of 99 ms. CSP assessed with 10% MVC of FDI. 10 stimuli delivered at 120% rMT. Duration of silent period measured from MEP onset, when EMG exceeded baseline level, until EMG activity reached/exceeded prestimulus baseline level for at least 50 ms **Functional** Difference in % accurate twitches between the first and last 10% of twitches	**TMS:** Arthritis: less SICI (0.98 ± 0.86 vs 0.57 ± 0.36) and greater SICF (5.4 ± 7.0 vs 2.1 ± 1.3) compared with healthy (both p = 0.03) No other significant differences in stimulus response, paired pulse, or cortical silent period **Functional:** No difference number of performed training twitches (arthritis: 755 ± 24; control: 749 ± 14; p = 0.3), magnitude of twitches (p = 0.6), or overall accuracy of training twitches (arthritis: 71% ± 21; control: 75% ± 21; P = 0.6) Arthritis: significantly lower number of accurate voluntary twitches (54% ± 30) in first 10% of trials compared with healthy (72% ± 42; p = 0.05) No difference in accuracy in last 10% of trials (arthritis: 72% ± 26; control: 72% ± 42; p = 0.98); however, change in accuracy from first to last 10%, reflecting skill learning, significantly greater for arthritis (18% ± 25) compared with Healthy (0% ± 43; p = 0.02)	Greater training-induced motor cortex reorganization was observed in people with hand pain due to arthritis compared to controls There was no evidence that these changes in cortical excitability are related to impaired motor function or skill learning	
Rittig-Rasmussen 2014a	To investigate i) neuroplastic changes of corticomotor pathways induced by neck training in patients with chronic neck or knee pain and in pain-free participants performing no training ii) the effect of pain and training on motor strength performance, motor learning capabilities, muscle fatigue, pain experience and pain catastrophizing	*Study population:* Neck pain n:20 F/M:6/14 Age: 29 (7) yrs Knee pain n:15 F/M: 5/10 Age: 27 (6) yrs Healthy controls n: 15 F/M: 3/12 Age: 25 (3.5) yrs *Study setting:* University laboratory *Study design:* Randomized study	**Experimental protocol** 2 experimental session (S1 and S2) **S1:** 1) TMS (baseline) 2) Task training 3) TMS (30 min and 1 h post-training) **S2:** 1) TMS (1-week post -training) **Task** Pain groups: 20 min upper trapezius training: elevating and lowering right shoulder 70 times in an ascending /descending movement path (moving a line displayed on the screen of a feedback system) with a load of 10% of maximal lifting capacity. *Total training time: 20 min* Healthy: no training **Outcomes measures** **TMS** Single pulse right trapezius muscle M1(target muscle) and right APB M1 (control muscle): MT set as minimum stimulus intensity that produced 5 discrete MEPs (> 50 uV). MT and TMS intensity were determined and stimuli were equivalent to 120–140% of the individual MT. Stimuli repeated approximately 4–6 times with increasing intensity until no further increase in amplitude was obtained and then 10 stimuli were delivered with 5-to 10-s ISI and averaged. Amplitudes and latencies of MEPs recorded at baseline and post task (30 min, 1 h, 7 days) from trapezius muscle and APB as control **Functional** Deviation from feedback curve between the first 5 and last 5 repetitions (of the total 70) only for the pain group	**TMS:** MEP amplitudes compared with baseline**:** Neck pain: significantly decreased at 30 min (p < 0.05), NS difference at 1 h and 1 week Knee pain: significantly increased at 30 min and 1 h (p < 0.01) but not after 1 week Healthy: NS at any time point Comparison between groups: Neck pain NS compared with other two groups (mean 1.57 mV ± 0.66) Healthy Higher mean amplitudes (mean 1.95 mV; 95%) compared to Knee pain (mean 1.31 mV; 95%) (p < 0.05) No difference for APB in any group. No difference in MEP latencies for trapezius or APB in any group over time Between baseline and 30 min, MEP amplitudes reduced: 18% in neck pain group, 28% in pain-free group; increased 36% in knee pain group **Functional:** Motor learning improved significantly in both pain groups. Neck pain + training 8.5% (p < 0.001) and knee pain + training 6.2%(p < 0.001)	Neck training reduced neuroplastic responsiveness of corticomotor pathways in neck pain patients in contrast to knee pain patients and pain-free participants	
Rittig-Rasmussen 2014b	To investigate the interaction between experimental neck pain and training and the effect on corticomotor excitability	*Study population:* Healthy population n: 52 F/M: 31/21 Age: 23 (2) yrs 20—32 *Study setting:* University laboratory *Study design:* Randomized study	**Experimental protocol** 2 experimental sessions (S1 and S2) (see Rittig-Rasmussen 2016a) **Experimental pain model** HS injection neck + training (n = 20) IS injection neck + trapezius training (n = 20) Control: HS injection neck, no training (n = 12) **Task** See training task Rittig-Rasmussen 2014a **Outcomes measures** **TMS/ functional** See Rittig-Rasmussen 2014a **Functional** See Rittig-Rasmussen 2014a Both training groups	**TMS:** Trapezius MEP amplitudes compared with baseline: HS + training: significantly decreased 30 min, 1 h, and 1 week (p < 0.0001) IS + training: significantly increased 30 min, 1 h, and 1 week (p < 0.0001) HS: significantly decreased 30 min and 1 h (p < 0.001) No difference for APB in any group MEP latencies compared with baseline: trapezius NS for any group (p = 0.075) APB: HS + training: significantly increased (0.19 ms) at 1 h (p < 0.01) IS + training: NS difference (p = 1.71) HS: significantly increased 1 h (p < 0.01) F**unctional:** Motor learning improved significantly in both groups HS + training: 4.5% (p < 0.001) IS + training: 6.2% (p < 0.001). NS between groups	The results infer that pain and concomitant training induce an enhanced and sustained inhibition of MEP amplitudes lasting for one week. Suggesting that motor training should be conducted in a pain-free manner as pain is an important factor in determining training-induced corticomotor excitability	
Schwenkreis 2011	To assess ICI during fatiguing muscle exercise in healthy humans and patients with Muscular dystrophy and Fibromyalgia syndrome to obtain insight into differential central mechanisms	*Study population:* Healthy population n: 23 F/M: 16/7 Age: 37.7 (11.5) yrs Muscular dystrophy n:23 F/M: 2/21 Age: 41 (10.4) yrs Fibromyalgia n:16 F/M: 14/2 Age: 48.7 (8.4) yrs *Study setting:* University laboratory *Study design:* Experimental study	**Experimental protocol** 1) TMS (baseline) 2) Task training 3) TMS (0- and 40-min post-training) **Task** Fatiguing muscle exercise by repeating right hand grip, 50% of maximum voluntary strength at a frequency of 1–2/s until they could no longer attain this force level **Outcome measures** **TMS** Single pulse right superficial flexor muscle of the forearm M1 (target muscle): Stimulus intensity adjusted to evoke a MEP of approximately 0.5 mv Paired pulse stimulation TMS for right superficial flexor muscle of the forearm M1 (target muscle): Test stimulus adjusted to evoke a MEP of approximately 0.5 mV; conditioning stimulus set at 80% of the individual MT. ISI at 2, 4, 10, and 15 ms. ICI at 2 ms and 4 ms, and ICF at 10 ms and 15 ms tested in resting muscle. CSP: to measure activity of subpopulation of intracortical inhibitory interneurons. CSP duration measured from the end of the MEP (onset of EMG suppression) until first re-occurrence of voluntary EMG activity	**TMS:** Healthy: significant difference MEP amplitudes between measures (p = 0.016). Significantly lower MEP amplitudes when comparing post-exercise vs. baseline (395.7 ± 303.8 uV vs. 534.8 ± 302.8 uV, p = 0.017) and with post40 (506.3 ± 363 uV p = 0.020) ICI differed significantly between measures (p = 0.019). Significantly decreased ICI, as indicated by an increased amplitude ratio post-exercise vs. baseline (50.0 ± 24.8% vs. 40.2 ± 15.4%, p = 0.029). ICF and CSP showed NS difference between measures Muscular dystrophy: NS between measures Fibromyalgia: ICI differed significantly between measurements (p = 0.032). Significant increase in ICI at post40 vs. post-exercise (49.2 ± 2 4.0% vs. 63.5 ± 36.0, p = 0.041). MEP amplitudes, ICF, and CSP were NS between measures	Fibromyalgia patients had lower ICI at baseline compared to healthy, but ICI increased after training	
Tsao 2010	To examine whether motor training can induce changes in motor cortical organization and whether such changes, if present, are associated with changes in postural activation of the trained muscles	*Study population:* Lower back pain Allocated to: *Training* n:10 F/M: 6/4 Age: 24 (8) yrs *Control* n:10 F/M: 5/5 Age: 23 (3) yrs *Study setting:* University laboratory *Study design:* Experimental study	**Experimental protocol** Two experimental sessions: before and after 2 weeks of task training 1) TMS + single rapid arm movements (baseline) 2) Task training (n = 10) or Control (n = 10) 3) TMS + single rapid arm movements (post-training) **Task and training** Skilled training at home (isolated voluntary contractions of transversus abdominis (TrA), control intervention of self-paced walking exercise for 2 weeks **Outcomes measures** **TMS** Single pulse TrA M1(target muscle): 5 stimuli at 120% AMT delivered over each scalp site during 10% MVC with an ISI of at least 5 s. If 120% AMT exceeded MSO, stimulation intensity for mapping was set to the MSO **Functional** Motor coordination assessed pre- and post-training using a rapid arm movement paradigm to induce postural challenges to the body	**TMS:** Motor cortical map: Training: anterior/medial shift over both hemispheres (p < 0.016) Control: No change in motor cortex representation (p > 0.57) No difference map volume between groups (p = 0.25) or after training (p = 0.30) MT not different between the left and right TrA (Double-cone coil: muscle p = 0.86; Figure-of-eight coil: muscle p = 0.85) No differences in MTs between groups prior to (p = 0.33), or pre- and post-training (p = 0.052). NS correlation between changes onset of TrA EMG and changes MTs (all r^2^ < 0.026, p > 0.35) **Functional:** Training induced earlier postural activation of TrA (interaction Time/Training: p < 0.001). No change in muscle activation following walking exercise (p = 0.86)	Motor skill training induced an anterior and medial shift in motor cortical representation of TrA in patients with back pain. This shift was associated with earlier postural activation of TrA	
Vallence 2013	To determine if neuroplasticity would be exaggerated in CTTH patients compared to healthy controls, which might explain (in part) the development of chronic pain in these individuals	*Study population:* CCTH: n:11 F/M: 6/5 Age: 35 (13.2) yrs Healthy controls: n:18 F/M:11/7 Age: 23 (8) yrs *Study setting:* University laboratory *Study design:* Experimental study	**Experimental protocol** Single experimental session 1) TMS (baseline) 2) Task training 3) TMS (0, 5-, 10-, 20- and 30-min post-training) **Task** 2 blocks of 225 thumb abduction (9 sub-blocks each with 25 abductions), with 5 min rest between the 2 blocks. Paced metronome 0.25 Hz *Total training time: 35 min including 5 min of break between two blocks* **Outcome measures** **TMS** rMT: before and 2 min after task training protocol Single pulse APB M1: Blocks of 15 single-pulse TMS trials with an inter-trial interval of 7 s (10%), at baseline and at 0, 5, 10, 20 and 30 min after the end of task training protocol **Functional** Peak acceleration of initial abduction movement after cue calculated for each trial (m/s^2^). Changes in mean peak acceleration with motor training	**TMS:** MEP amplitude after training: No significant change in rMT for CTTH or controls (both p > 0.05). Significant effect of time for controls (p < 0.05) but not CTTH (p > 0.05); significant increase in MEP amplitude after motor training for controls Controls: MEP amplitude was significantly increased 10- and 20-min post-training (p < 0.05). 30 min after training, MEP amplitude had returned to baseline levels (p > 0 0.5) **Functional:** Greater increase in acceleration with motor training in healthy controls than CTTH; controls significantly greater mean peak acceleration in sub-blocks 3–9 and 12–18 (both p < 0.05)	Individuals with CTTH showed significantly less motor learning on the training task than healthy controls, suggesting a deficit in use-dependent neuroplasticity within networks responsible for task performance in patients with CTTH	

CBI, cerebellar inhibition; MA, motor acquisition task; TMS, transcranial magnetic stimulation; FDI, First dorsal interosseus; MEP, Motor Evoked potential; rMT, Resting motor threshold; RCT, Randomized clinical control trial; TPT, Tongue-protrusion task; AUC, Area under the curve; NRS, Numeric pain rating scale; IO, input–output; APB, abductor pollicis brevis; NGF, Nerve growth factor; DOMS, Delayed onset muscle soreness; PPT, Pressure pain thresholds; CoG, Center of gravity; ECERB, extensor carpi radialis brevis; MSO, maximum stimulator output; HS, Hypertonic saline; IS, Isotonic saline; APA, Anticipatory postural activation; RPMS, Repetitive peripheral magnetic stimulation; MF, multifidus muscle; VAS, Visual analog scale; AMT, Active motor threshold; MSO, Maximal stimulation output; ICI, Intracortical inhibition; ICF, intracortical facilitation; tDCS, transcranial direct current stimulation; QOL, Quality of Life, AE, Aerobic exercise ICI: intracortical inhibition; ISI, interstimulus intervals; CSP, Cortical silent period; APB, Abductor pollicis brevis; CTTH, Chronic tension-type headache; PB, abductor pollicis brevis; MT, Motor threshold; SICI, Short interval intracortical inhibition; SICF, Short interval intracortical facilitation; LICI, Long interval intracortical inhibition; LICF, Long-interval intracortical inhibition facilitation

The majority of the included articles focused on chronic pain conditions (n = 10) (Baarbe et al., 2018; Hoeger Bement et al., 2014; Masse-Alarie et al., 2016, 2017b; Mendonca et al., 2016; Parker et al., 2017; Rittig-Rasmussen et al., 2014a; Schwenkreis et al., 2011; Tsao et al., 2010; Vallence et al., 2013), and the remaining articles focused on acute pain in healthy individuals (n = 7) (Boudreau et al., 2007; Dancey et al., 2019; De Martino, Petrini, et al., 2018; De Martino, Zandalasini, et al., 2018; Ingham et al., 2011; Mavromatis et al., 2017; Rittig-Rasmussen et al., 2014b). The chronic pain conditions included headache (n = 1), pain in the neck region (n = 2), lower back pain (n = 3), painful hand arthritis (n = 1) and fibromyalgia (n = 3).

Acute pain was induced by either: i) applying capsaicin cream (n = 3) intraorally (Boudreau et al., 2007), on the right thumb (Mavromatis et al., 2017), or on the dominant elbow (Dancey et al., 2019); ii) by injecting hypertonic saline (n = 2) into the right first dorsal interosseous muscle (Ingham et al., 2011) or the right side of the neck (Rittig-Rasmussen et al., 2014b); or iii) eccentric exercise to induce delayed onset muscle soreness (n = 2): in wrist extensor muscles (De Martino, Petrini, et al., 2018; De Martino, Zandalasini, et al., 2018).

Regarding the training paradigms, only one study focused on the trigeminally-innervated region and involved training in a tongue-protrusion task (Boudreau et al., 2007). The remaining studies focused mainly on the spinally-innervated upper extremities (hands/arms) and most often involved training in different finger tasks (n = 6). These finger tasks included: typing letter sequences on a keyboard (Baarbe et al., 2018), tracing sinusoidal waves on a touchpad with dominant thumb (Dancey et al., 2019), brisk movements with the index finger in the opposite direction to the twitches evoked by TMS (Ingham et al., 2011), pinching a force transducer (Mavromatis et al., 2017), voluntary finger twitching (Parker et al., 2017) and thumb abduction in response to an auditory cue (Vallence et al., 2013).

Six of the seven acute pain studies (Boudreau et al., 2007; Dancey et al., 2019; De Martino, Petrini, et al., 2018; De Martino, Zandalasini, et al., 2018; Mavromatis et al., 2017; Rittig-Rasmussen et al., 2014b) and five of the ten chronic pain studies (Hoeger Bement et al., 2014; Masse-Alarie et al., 2016; Rittig-Rasmussen et al., 2014a; Schwenkreis et al., 2011; Vallence et al., 2013) showed that acute and chronic pain impede trainings-induced functional neuroplasticity otherwise observed in the primary motor cortex (e.g., increased corticomotor excitability).

In terms of motor performance, only five of the seven studies that induced acute pain evaluated subsequent changes in motor performance (Boudreau et al., 2007; Dancey et al., 2019; Ingham et al., 2011; Mavromatis et al., 2017; Rittig-Rasmussen et al., 2014b). Two of these five studies (Boudreau et al., 2007; Ingham et al., 2011) showed that pain in the region being trained (i.e., tongue and finger, respectively) had a negative effect on the training-induced motor performance gain (i.e., tongue protrusion and finger abduction, respectively). The remaining three studies (Dancey et al., 2019; Mavromatis et al., 2017; Rittig-Rasmussen et al., 2014b) did not demonstrate any differences between pain and control groups.

For the chronic pain studies, only five of the ten studies (De Martino, Petrini, et al., 2018; De Martino, Zandalasini, et al., 2018; Hoeger Bement et al., 2014; Mendonca et al., 2016; Schwenkreis et al., 2011) evaluated motor performance in comparison to pain-free control groups. Four of these studies did not show any conclusive findings whereas one study on chronic headache showed greater improvement in a thumb abduction motor task in the pain-free control group (Vallence et al., 2013).

### Risk of bias assessment

The risk of bias as assessed with the use of NOS is presented in Table 4. Lower scores were generally associated with study design and often with lack of inadequate control groups. Therefore, we added a modified score for internal control groups. For the assessment with the TMS checklist the average quality score was 72% (range 54—90%), for reported factors and 65% (range 38 – 83%) for controlled factors (Table 5). All studies scored over 50% for factors that should be reported and only three studies scored less than 50% (Baarbe et al., 2018; Hoeger Bement et al., 2014; Mendonca et al., 2016) for factors that should be controlled. However, none of the included studies were rated as having an overall high risk of bias and all studies were retained for the qualitative synthesis.

**Table 4:** Risk of bias in included studies (n = 17) assessed with a modified Newcastle–Ottawa Scale for case–control studies

First author Year	Selection				Comparability		Exposure			Total
Assessment Category	S1	S2	S3	S4	C1a	C1b	E1	E2	E3	
Experimental pain in healthy (n = 7)										
Boudreau 2007*	★	**-**	**-**	(★)	★	(★)	★	(★)	(★)	3 (4)
Dancey 2019	★	**-**	★	★	★	★	★	★	★	8
De Martino 2018a*	★	**-**	**-**	(★)	(★)	(★)	★	(★)	**-**	2 (4)
De Martino 2018b*	★	**-**	**-**	(★)	(★)	(★)	★	(★)	**-**	2 (4)
Ingham 2011*	★	**-**	**-**	(★)	(★)	(★)	★	(★)	**-**	2 (4)
Mavromatis 2017	★	**-**	**-**	★	★	★	★	★	★	7
Rittig-Rasmussen 2014b*	★	**-**	(★)	(★)	(★)	(★)	★	(★)	(★)	2 (6)
Chronic pain population (n = 10)										
Baarbe 2018	★	**-**	**-**	★	★	★	★	★	**-**	6
Hoeger Bement 2014*	★	**-**	**-**	(★)	(★)	(★)	★	(★)	★	2 (4)
Massé-Alaire 2017b*	★	**-**	(★)	(★)	★	★	★	★	★	6 (2)
Massé-Alaire 2016*	★	**-**	(★)	(★)	★	★	★	★	**-**	5 (2)
Mendonca 2016	★	**-**	★	★	★	★	★	★	**-**	7
Parker 2017	★	★	★	★	★	★	★	★	★	9
Rittig-Rasmussen 2014a	★	**-**	★	★	★	★	★	★	**-**	7
Schwenkreis 2011	★	**-**	**-**	★	★	**-**	★	★	★	6
Tsao 2010	★	**-**	**-**	★	★	★	★	★	**-**	6
Vallence 2013	★	**-**	**-**	★	★	★	★	★	**-**	6

S – Selection of case and control; C – Comparability of cases vs control; E – Exposure

S1: Case Definition; S2: Case Representativeness; S3: Control Selection; S4: Control Definition. C1a: Age; C1b: Other factors. E1: Assessment; E2: Same method cases and controls; E3: Nonresponse rate

^*^Please note that per the definitions of the criteria in the Newcastle–Ottawa scale studies without a control group cannot achieve scores for items S3, S4, C1a, C1b, E2 and E3. We have therefore modified the scale by adding a score (in brackets) for internal control groups

**Table 5:** Quality assessment of included articles (n = 17) with the TMS checklist

First author Year	Reported				Controlled			
	Yes	No	N/A	% Yes	Yes	No	N/A	% Yes
Baarbe 2018	15	9	6	63	11	12	7	48
Boudreau 2007	19	6	5	76	16	9	5	64
Dancey 2019	19	5	6	79	17	7	6	71
De Martino 2018a	17	8	5	68	14	11	5	56
De Martino 2018b	18	7	5	72	18	7	5	72
Hoeger Bement 2014	13	11	6	54	9	15	6	38
Ingham 2011	19	7	5	72	18	7	5	72
Masse Alaire 2017b *	23	6	1	79	23	6	1	79
Masse Alaire 2016 *	22	7	1	76	22	7	1	76
Mavromatis 2017 *	20	8	2	71	19	9	2	68
Mendonca 2016 *	15	13	2	54	13	15	2	46
Parker 2017 *	26	3	1	90	24	5	1	83
Rittig-Rasmussen 2014a	20	5	5	80	17	8	5	68
Rittig-Rasmussen 2014b	19	6	5	76	16	9	5	64
Schwenkreis 2011*	18	10	2	64	18	10	3	64
Tsao 2010	17	7	6	71	16	8	6	67
Vallence 2013	18	6	6	75	17	7	6	71
Mean	19	7	4	72	17	9	4	65

^*^ Paired pulse. otal number of possible reported/controlled items are 26 for single pulse TMS, and 29 for paired pulse TMS

### Meta-analysis

When all included papers were assessed for the possibility of a quantitative analysis, there was a lack of studies reporting central and dispersion measures for comparable outcomes. Two studies from the same research group (Masse-Alarie et al., 2016, 2017b) in chronic back pain patients reported short intracortical inhibition (SICI) and facilitation (SICF) (before and after intervention). The same studies, together with one study on acute pain in healthy individuals (De Martino, Zandalasini, et al., 2018), presented data on MEP amplitudes and silent periods. Based on this, it was not deemed suitable to carry out a meta-analysis but instead a qualitative synthesis of main findings is presented (Table 6).

**Table 6:**
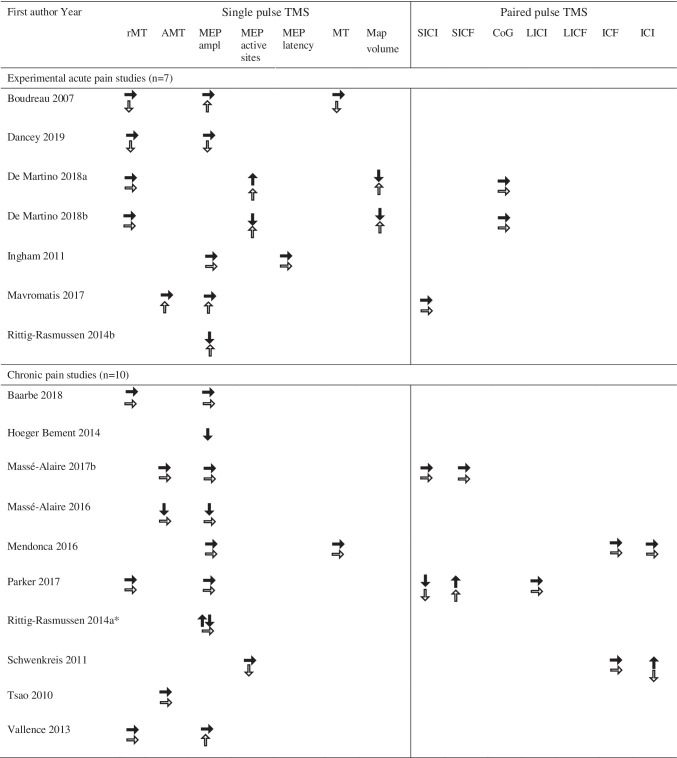
Results reported for pain groups (black arrows) and control groups (white arrows) as no changes (→), increase (↑) and decrease (↓) for the included studies (n = 17)

rMT, Resting motor threshold; AMT, Active motor threshold; MEP, Motor evoked potential; MT, Motor threshold; SICI, Short interval intracortical inhibition; SICF, Short interval intracortical facilitation; CoG, Center of gravity; LICI, Long interval intracortical inhibition; LICF, Long-interval intracortical inhibition facilitation ICF, Intracortical facilitation; ICI, intracortical inhibition

## Discussion

### Pain, training, and neuroplasticity

This systematic review included 7 studies investigating the impact of experimental acute pain in pain-free participants, and 10 studies investigating the effects of chronic pain on corticomotor excitability of the muscle in pain and the associated training-induced motor performance gain. The main findings suggest that both acute and chronic pain may impede training-induced neuroplasticity.

The studies included in the present review were heterogeneous with regard to several aspects of study designs. One aspect to consider is that the selected cases included in the studies represented a wide range of chronic pain conditions and different acute pain protocols, manifested at different regions of the body. The chronic pain conditions included either widespread pain in multiple anatomical locations (i.e., fibromyalgia), regional pain in the cervical, spinal, or lumbar regions, or localized pain in the hand, head and facial regions. The acute pain protocols ranged from application of a topical capsaicin cream or hypertonic saline injection to the target muscle or nearby area, to injections of a nerve growth factor, eccentric exercises to provoke delayed onset muscle soreness, or a combination of the last two. Furthermore, the training tasks differed between studies. Some studies were simpler and involved only one (i.e., one-dimensional) or a few muscles, whereas other studies were more complex with tasks involving coordinated activation of multiple muscles and muscle groups as well as being more cognitively demanding. The range of training tasks included some that can be presumed, at least in part, to utilize already existing motor pattern skills such as typing (Baarbe et al., 2018), whereas other motor tasks can be viewed as more novel motor patterns, e.g., tracing an external target utilizing shoulder muscles (Rittig-Rasmussen et al., 2014a, 2014b). Other studies incorporated more fatiguing or strength-demanding tasks (De Martino, Petrini, et al., 2018; De Martino, Zandalasini, et al., 2018; Hoeger Bement et al., 2014; Mavromatis et al., 2017), thereby putting additional functional demands on muscles, muscle groups and the corticospinal tract.

The large variation in the duration, repetition, intensity and type of training performed, each of which can impact the neuroplastic changes occurring in the brain, is consistent with the time, intensity, repetition and specificity principles of neuroplasticity (Avivi-Arber & Sessle, 2018; Kleim & Jones, 2008). This relates to the question “when is enough enough?” or maybe a ceiling effect for pain conditions. It has been shown that neuroplastic changes are more pronounced in skills training (Pascual-Leone et al., 1995), as opposed to fatiguing and strength training exercises (Remple et al., 2001) and that exercise-induced fatigue may even reduce neuroplasticity (Wang et al., 2020). In the present review, several of the primary studies included training protocols either designed to induce fatigue (De Martino, Petrini, et al., 2018; De Martino, Zandalasini, et al., 2018; Hoeger Bement et al., 2014; Mendonca et al., 2016; Schwenkreis et al., 2011), or protocols that may have induced fatigue due to the length of training sessions or the load applied (Boudreau et al., 2007; Parker et al., 2017; Rittig-Rasmussen et al., 2014a, 2014b; Vallence et al., 2013). In addition to the duration of individual sessions, the number of sessions may also affect the overall effects from training, consistent with the ‘repetition’ principle of neuroplasticity.

Consistent with the ‘timing’ principle of neuroplasticity (Avivi-Arber & Sessle, 2018; Kleim & Jones, 2008), the time between training and TMS measurements should be considered in relation to immediate versus more long-lasting neuroplastic changes. Time is an important factor since different forms and different directions of neuroplastic changes occur at different points of time after motor training (Avivi-Arber & Sessle, 2018; Kleim & Jones, 2008). Whereas most studies performed TMS measurement to assess neuroplastic changes immediately after training (i.e., within one-hour timeframe), several studies also explored training-induced neuroplastic changes more thoroughly by performing multiple TMS measurements within the first 15-min (Ingham et al., 2011; Parker et al., 2017; Vallence et al., 2013) or first hour (Rittig-Rasmussen et al., 2014a, 2014b; Schwenkreis et al., 2011; Vallence et al., 2013) following training. Two studies also investigated possible long term effects after one week of training (Rittig-Rasmussen et al., 2014a, 2014b) and found a long lasting inhibition of corticomotor excitability in healthy subjects who performed neck exercises in the presence of acute neck pain (Rittig-Rasmussen et al., 2014b). Therefore, it is noteworthy in the context of training, that pain can induce fast-onset and long-lasting neuroplastic changes manifested as decreased corticomotor excitability. Clearly, there are several important training-related parameters that need to be taken into consideration before a more comprehensive understanding of the effects of pain on training-induced corticomotor excitability can be obtained.

The wide range of pain conditions discussed above can explain variations across the studies in the expression of pain sensations in terms of intensity and quality of pain, as well as temporal aspects of pain such as timing of peak intensity and duration. Consistent with the ‘specificity’ principle of neuroplasticity (Avivi-Arber & Sessle, 2018; Kleim & Jones, 2008), these variations in pain characteristics may explain differences in neuroplastic changes between selected cases and controls within and across studies.

Additionally, age is a factor that should be considered as aging is associated with different processes, e.g., physiological degradation and neuronal atrophy, resulting in declines in sensorimotor control and performance. There was a large mean age range from 21.1 years (Baarbe et al., 2018) to 72.0 years (Parker et al., 2017) for the studies evaluating the effect of chronic pain on corticomotor excitability, whereas for the studies that induced acute pain in pain-free participants the mean age ranged only from 20.2 years (Dancey et al., 2019) to 26.5 years (Mavromatis et al., 2017). However, no significant effects attributed to age were found for any of the included studies. Another subject related factor worth taking into consideration is that of gender. All studies reported varying ratios of male and female participants. According to the TMS checklist gender is a factor that only should be reported and not controlled, which was the case for all included studies.

As mentioned earlier, most studies incorporated training that targeted painful areas, either through acute pain or by the presence of localised chronic pain conditions. There was however a number of studies that examined the more global effects, from chronic tension type headaches (Vallence et al., 2013), neck pain (Baarbe et al., 2018), back pain (Tsao et al., 2010) or fibromyalgia (Schwenkreis et al., 2011) in hand or arm training or from knee pain in neck training (Rittig-Rasmussen et al., 2014a). The last of these studies investigated neck training in three groups; neck pain, knee pain and controls, and demonstrated significantly reduced corticomotor excitability of neck muscles as assessed by MEP amplitudes in the neck pain, but not in the knee pain or control groups (Rittig-Rasmussen et al., 2014a). This finding is in accordance with a lack of difference in MEP amplitudes between healthy controls and a neck pain group following a typing task (Baarbe et al., 2018), and between healthy controls and a fibromyalgia group following a hand grip task (Schwenkreis et al., 2011). This finding strongly indicates that it may not be pain in general but pain in a relevant region for the motor task that determines the impact on training-induced neuroplasticity. In contrast, reduced motor learning in a thumb abduction task was reported for headache patients compared to healthy controls (Vallence et al., 2013), indicating that the relationship between pain location and trained regions may be dependent not only on the location but also on the type of pain condition.

There are several factors to consider when comparing the chronic pain and acute pain groups of the studies included in the present review. One aspect is that in the chronic pain conditions, and in most of the acute pain conditions, motor skill acquisition training and motor skill acquisition occurred in the presence of pain. In contrast, in two studies of acute pain, motor training occurred in the absence of pain but the motor skill acquisition was evaluated in the presence of pain (De Martino, Petrini, et al., 2018; De Martino, Zandalasini, et al., 2018). In a systematic review based on 43 studies that evaluated motor cortex excitability in chronic pain conditions, Parker et al. found that chronic pain conditions can induce a range of motor cortex neuroplastic changes that vary across studies there was inconsistency for most outcome measures. Among the changes were reduced duration of silent period and SICI together with enhanced SICF. There were also indications that these effects were more pronounced in populations with neuropathic pain (Parker et al., 2016). Most of the included studies were however based on migraine populations, thereby representing a pain condition with complex pathophysiology specifically related to the trigeminal region.

### Functional aspects

Another factor to consider when comparing exercise in the presence of chronic pain versus acute pain is the “salience principle” (Kleim & Jones, 2008). The salience principle describes the brain's attention to any input, and in the context of exercise can be seen as increased attention to a task being practiced. Patients with chronic pain may have such a cognitive incentive when exercising, due to perceived or expected healing or analgesic effects. In acute pain, however, exercise probably has no cognitive incentive, but rather may have an “interference” effect, e.g., worsening the acute pain, thereby resulting in non-salient exercise. This is supported by the increased representation in the motor cortex from salient exercise compared to non-salient exercise (Stefan et al., 2004). It has been suggested that this may be explained in part by the increase of acetylcholine in the cortex in salient exercises compared to non-salient exercises (Meintzschel & Ziemann, 2006).

With regard to the functional outcomes, there was a large variety also here with regard to reported outcomes, and five of the ten chronic pain studied (De Martino, Petrini, et al., 2018; De Martino, Zandalasini, et al., 2018; Hoeger Bement et al., 2014; Mendonca et al., 2016; Schwenkreis et al., 2011) and six of the seven acute pain studies (Boudreau et al., 2007; Dancey et al., 2019; De Martino et al., 2018a; De Martino et al., 2018b; Mavromatis et al., 2017; Rittig-Rasmussen et al., 2014b), did not report specific functional outcomes. Different types of percentage performance score were reported in relation to tracking tasks (Boudreau et al., 2007; Rittig-Rasmussen et al., 2014a, 2014b) and accuracy (Baarbe et al., 2018; Mavromatis et al., 2017; Parker et al., 2017) sometimes combined with response time or speed of movement (Baarbe et al., 2018; Mavromatis et al., 2017). In general, performance improved with training regardless of presence of pain, although some performance scores were affected in pain groups compared to pain-free controls (Boudreau et al., 2007; Vallence et al., 2013).

### Quality assessment

As per our Prospero protocol we carried out a risk of bias assessment utilizing two instruments. The first instrument, NOS, covers domains for selection of participants, comparability between groups, and exposure, and is the recommended instrument for case–control studies. A general finding across all studies was that the study populations were often convenience samples and therefore not truly representative of the populations under study. Furthermore, our assessment was based on the specific aim to evaluate neuroplasticity after training in the presence of pain. With regard to study design, studies without a control group can by definition only be scored on three items in the NOS and we therefore added a modified score for studies with internal control groups. Furthermore, the quality scores for some of the included studies could be higher if they had been assessed in relation to the specific aim of the respective studies. We therefore regard the results from the assessment with NOS to be of limited value in the present review, given that many studies were based on intraindividual comparisons, before and after training, and not on comparisons between groups. We also carried out an assessment with the TMS checklist which was introduced in 2012 (Chipchase et al., 2012). This instrument was developed based on a 2-round international Delphi process with 42 participants resulting in a 30-item checklist covering domains of participant characteristics, TMS protocol and analysis. The overall mean scores for the primary studies in the present review of 72 and 68% for reported and controlled factors, respectively, indicate a moderate methodological quality and are in line with other reviews utilizing this checklist. Items in the checklist that were reported to a less degree included prescribed medication and history of repetitive motor activity for participants, participants’ attention during testing, and size of unconditioned MEP in the target muscles. These findings are also in accordance with other reviews (Parker et al., 2016; Rosso & Lamy, 2018) indicating that the primary studies in the present review have a methodological quality similar to other recent systematic reviews.

### Neurophysiological aspects

The main goal of many neurorehabilitation regimes is to promote neuroplasticity at the subcortical and cortical levels, such that long-lasting and beneficial alterations in motor control strategies can be achieved (Gabriel et al., 2006). Novel motor skill training, in contrast to passive assistance or repetitions of general exercise (strength training), has been associated with improvements in task performance and increased representation of the trained muscle in the motor cortex (Kothari et al., 2013; Svensson et al., 2003). At this point in time, in terms of evaluating the combined effect of pain and motor training on neuroplasticity, there was not sufficient data available that could be extracted from the primary studies and grouped according to outcome measures to carry out a meta-analysis. However, from the qualitative synthesis we can conclude that both acute pain and chronic pain may impede training-induced neuroplasticity which may have implications for motor learning and performance during rehabilitation following injury or disease. Training-induced neuroplasticity has been shown to occur rapidly and to continually evolve with more training (Koeneke et al., 2006; Svensson et al., 2003). It is therefore reasonable to assume that presence of pain may impede plasticity induced by long-term training in a similar manner to short-term training, as reported in the present review.

The results from the present review are in line with the principle that pain, both acute and chronic, is not purely a sensory process but that pain networks interact with other complex networks in cerebral structures including, but not limited to, the primary motor cortex (M1), thalamus and prefrontal cortices. Such interactions are used for example to initiate and modulate actions to avoid or reduce pain. The possible interaction between pain and the M1 can influence training-induced neuroplasticity and some of the findings in the present review may to some extent indicate the neurophysiological processes involved. Single pulse TMS measure outcomes, such as a decrease in rMT, were reported in control conditions but negated by pain. This may indicate increased excitability in a central core region of neurons in M1. On the other hand, an increase in MEPs may imply involvement of additional neurons in other regions (Hallett et al., 1999). Variations in paired pulse TMS outcomes such as ICI represent changes to the inhibitory cortical networks primarily regulated by the chief inhibitory neurotransmitter GABA (Hallett et al., 1999). Reduced activity in these inhibitory cortical networks is an indication of enhanced neuroplasticity but may be negated in the presence of pain. Furthermore, pain may also affect performance due to movement related pain or kinesiophobia directly resulting in impaired motor output (Hodges & Smeets, 2015; Hodges & Tucker, 2011). Consequently, the performed training of a specific motor skill is hampered which indirectly impedes the associated training-induced neuroplasticity.

### Summary

The present study has shown that both acute pain and chronic pain may impede training-induced functional neuroplasticity manifested as decreased corticomotor excitability as defined by TMS. Overall, the findings reflect the many aspects of human neuroplasticity, that cannot be encapsulated by a single outcome measure. It should be acknowledged that other brain imaging techniques such as structural and functional magnetic resonance imaging (sMRI, fMRI), electroencephalography (EEG) and magnetoencephalography (MEG) can provide complementary information that can help identify neural correlates underlying a particular neuroplastic brain change and associated motor behaviour. This information is important for developing better rehabilitative training approaches that adequately manage pain and facilitate adaptive neuroplasticity and improved motor performance.

## Supplementary Information

Below is the link to the electronic supplementary material.

Supplementary file1 (PDF 614 kb)

## Data Availability

Not applicable it is a systematic review, but the data can be made available by the authors if requested.

## References

[CR1] Akhter R, Benson J, Svensson P, Nicholas MK, Peck CC, Murray GM (2014). Experimental jaw muscle pain increases pain scores and jaw movement variability in higher pain catastrophizers. Journal of Oral & Facial Pain and Headache.

[CR2] Avivi-Arber L, Sessle BJ (2018). Jaw sensorimotor control in healthy adults and effects of ageing. Journal of Oral Rehabilitation.

[CR3] Baarbe JK, Yielder P, Haavik H, Holmes MWR, Murphy BA (2018). Subclinical recurrent neck pain and its treatment impacts motor training-induced plasticity of the cerebellum and motor cortex. PLoS ONE.

[CR4] Bank PJ, Peper CE, Marinus J, Beek PJ, van Hilten JJ (2013). Motor consequences of experimentally induced limb pain: A systematic review. European Journal of Pain.

[CR5] Boudreau S, Romaniello A, Wang K, Svensson P, Sessle BJ, Arendt-Nielsen L (2007). The effects of intra-oral pain on motor cortex neuroplasticity associated with short-term novel tongue-protrusion training in humans. Pain.

[CR6] Bradnam L, McDonnell M, Ridding M (2016). Cerebellar intermittent theta-burst stimulation and motor control training in individuals with cervical dystonia. Brain Sciences.

[CR7] Chang, Y. (2014). Reorganization and plastic changes of the human brain associated with skill learning and expertise. *Frontiers in Human Neuroscience*, 8. 10.3389/fnhum.2014.00035 10.3389/fnhum.2014.00035 PMC3912552 24550812

[CR8] Chipchase L, Schabrun S, Cohen L, Hodges P, Ridding M, Rothwell J, Taylor J, Ziemann U (2012). A checklist for assessing the methodological quality of studies using transcranial magnetic stimulation to study the motor system: An international consensus study. Clinical Neurophysiology.

[CR9] Daligadu J, Haavik H, Yielder PC, Baarbe J, Murphy B (2013). Alterations in cortical and cerebellar motor processing in subclinical neck pain patients following spinal manipulation. Journal of Manipulative and Physiological Therapeutics.

[CR10] Dancey, E., Yielder, P., & Murphy, B. (2019). The Interactive Effect of Tonic Pain and Motor Learning on Corticospinal Excitability. *Brain Sci*, *9*(3). 10.3390/brainsci9030063 10.3390/brainsci9030063 PMC6468489 30884779

[CR11] De Martino E, Petrini L, Schabrun S, Graven-Nielsen T (2018). Cortical Somatosensory Excitability Is Modulated in Response to Several Days of Muscle Soreness. The Journal of Pain.

[CR12] De Martino E, Zandalasini M, Schabrun S, Petrini L, Graven-Nielsen T (2018). Experimental muscle hyperalgesia modulates sensorimotor cortical excitability, which is partially altered by unaccustomed exercise. Pain.

[CR13] Dettmers C, Adler T, Rzanny R, van Schayck R, Gaser C, Weiss T, Miltner WH, Bruckner L, Weiller C (2001). Increased excitability in the primary motor cortex and supplementary motor area in patients with phantom limb pain after upper limb amputation. Neuroscience Letters.

[CR14] Duchateau J, Semmler JG, Enoka RM (2006). Training adaptations in the behavior of human motor units. Journal of Applied Physiology (1985).

[CR15] Gabriel DA, Kamen G, Frost G (2006). Neural adaptations to resistive exercise: Mechanisms and recommendations for training practices. Sports Medicine (auckland, n. z.).

[CR16] Gurevich M, Kohn PM, Davis C (1994). Exercise-induced analgesia and the role of reactivity in pain sensitivity. Journal of Sports Sciences.

[CR17] Hallett, M., Chen, R., Ziemann, U., & Cohen, L. G. (1999). Reorganization in motor cortex in amputees and in normal volunteers after ischemic limb deafferentation. *Electroencephalogr Clin Neurophysiol Suppl*, *51*, 183–187. https://www.ncbi.nlm.nih.gov/pubmed/10590950 10590950

[CR18] Hellmann D, Giannakopoulos NN, Blaser R, Eberhard L, Rues S, Schindler HJ (2011). Long-term training effects on masticatory muscles. Journal of Oral Rehabilitation.

[CR19] Hodges PW, Smeets RJ (2015). Interaction Between Pain, Movement, and Physical Activity Short-term Benefits, Long-term Consequences, and Targets for Treatment. Clinical Journal of Pain.

[CR20] Hodges PW, Tucker K (2011). Moving differently in pain: A new theory to explain the adaptation to pain. Pain.

[CR21] HoegerBement M, Weyer A, Yoon T, Hunter S (2014). Corticomotor Excitability During a Noxious Stimulus Before and After Exercise in Women With Fibromyalgia. Journal of Clinical Neurophysiology.

[CR22] Ingham D, Tucker KJ, Tsao H, Hodges PW (2011). The effect of pain on training-induced plasticity of the corticomotor system. European Journal of Pain.

[CR23] Kleim JA, Jones TA (2008). Principles of experience-dependent neural plasticity: Implications for rehabilitation after brain damage. Journal of Speech, Language, and Hearing Research.

[CR24] Koeneke S, Lutz K, Herwig U, Ziemann U, Jäncke L (2006). Extensive training of elementary finger tapping movements changes the pattern of motor cortex excitability. Experimental Brain Research.

[CR25] Kosek E, Roos EM, Ageberg E, Nilsdotter A (2013). Increased pain sensitivity but normal function of exercise induced analgesia in hip and knee osteoarthritis–treatment effects of neuromuscular exercise and total joint replacement. Osteoarthritis Cartilage.

[CR26] Kothari M, Svensson P, Jensen J, Kjærsgaard A, Jeonghee K, Nielsen JF, Ghovanloo M, Baad-Hansen L (2013). Training-induced cortical plasticity compared between three tongue-training paradigms. Neuroscience.

[CR27] Krause P, Forderreuther S, Straube A (2006). TMS motor cortical brain mapping in patients with complex regional pain syndrome type I. Clinical Neurophysiology.

[CR28] Lohse KR, Wadden K, Boyd LA, Hodges NJ (2014). Motor skill acquisition across short and long time scales: A meta-analysis of neuroimaging data. Neuropsychologia.

[CR29] Massé-Alarie, H., Louis-David, B., Preuss, R., Schneider, C. (2015). Multifidus voluntary training versus hip extension exercises in chronic low back pain: effects on clinical outcomes and underlying corticomotor function. *Physiotherapy*, 101e9609–e961. 10.1016/j.physio.2015.03.1814

[CR30] Masse-Alarie H, Beaulieu LD, Preuss R, Schneider C (2016). Influence of paravertebral muscles training on brain plasticity and postural control in chronic low back pain. Scandinavian Journal of Pain.

[CR31] Massé-Alarie H, Beaulieu LD, Preuss R, Schneider C (2017). The side of chronic low back pain matters: evidence from the primary motor cortex excitability and the postural adjustments of multifidi muscles. Experimental Brain Research.

[CR32] Masse-Alarie, H., Beaulieu, L. D., Preuss, R., & Schneider, C. (2017b). Repetitive peripheral magnetic neurostimulation of multifidus muscles combined with motor training influences spine motor control and chronic low back pain. *Clinical Neurophysiology,**128*(3), 442–453. 10.1016/j.clinph.2016.12.020 10.1016/j.clinph.2016.12.020 28160750

[CR33] Mavromatis, N., Neige, C., Gagne, M., Reilly, K. T., & Mercier, C. (2017). Effect of Experimental Hand Pain on Training-Induced Changes in Motor Performance and Corticospinal Excitability. *Brain Sci*, *7*(2). 10.3390/brainsci7020015 10.3390/brainsci7020015 PMC5332958 28165363

[CR34] McCambridge, A. B., Bradnam, L. V. (2018). S63. Cerebellar stimulation for adults with cervical dystonia: A tDCS study. *Clinical Neurophysiology, 129e165*. 10.1016/j.clinph.2018.04.423

[CR35] Meintzschel F, Ziemann U (2006). Modification of practice-dependent plasticity in human motor cortex by neuromodulators. Cerebral Cortex.

[CR36] Mendonca, M. E., Simis, M., Grecco, L. C., Battistella, L. R., Baptista, A. F., & Fregni, F. (2016). Transcranial Direct Current Stimulation Combined with Aerobic Exercise to Optimize Analgesic Responses in Fibromyalgia: A Randomized Placebo-Controlled Clinical Trial. *Frontiers in Human Neuroscience*, 10. 10.3389/fnhum.2016.00068 10.3389/fnhum.2016.00068 PMC4785149 27014012

[CR37] Michelini LC, Stern JE (2009). Exercise-induced neuronal plasticity in central autonomic networks: Role in cardiovascular control. Experimental Physiology.

[CR38] Moher D, Liberati A, Tetzlaff J, Altman DG, Group P (2009). Preferred reporting items for systematic reviews and meta-analyses: the PRISMA statement. Journal of Clinical Epidemiology.

[CR39] Parker, R., Lewis, G., Rice, D., & McNair, P. (2016). Is Motor Cortical Excitability Altered in People with Chronic Pain? A Systematic Review and Meta-Analysis. *Brain Stimulation,**4*, 488–500. 10.1016/j.brs.2016.03.020 10.1016/j.brs.2016.03.020 27133804

[CR40] Parker, R., Lewis, G., Rice, D., & McNair, P. (2017). The Association between Corticomotor Excitability and Motor Skill Learning in People with Painful Hand Arthritis. *The Clinical Journal of Pain*, 33(3), 222–230. 10.1097/ajp.0000000000000392 10.1097/AJP.0000000000000392 27258992

[CR41] Pascual-Leone A, Nguyet D, Cohen LG, Brasil-Neto JP, Cammarota A, Hallett M (1995). Modulation of muscle responses evoked by transcranial magnetic stimulation during the acquisition of new fine motor skills. Journal of Neurophysiology.

[CR42] Pascual-Leone A, Amedi A, Fregni F, Merabet LB (2005). THE PLASTIC HUMAN BRAIN CORTEX. Annual Review of Neuroscience.

[CR43] Remple MS, Bruneau RM, VandenBerg PM, Goertzen C, Kleim JA (2001). Sensitivity of cortical movement representations to motor experience: evidence that skill learning but not strength training induces cortical reorganization. Behavioural Brain Research.

[CR44] Rittig-Rasmussen B, Kasch H, Fuglsang-Frederiksen A, Jensen TS, Svensson P (2013). Specific neck training induces sustained corticomotor hyperexcitability as assessed by motor evoked potentials. Spine.

[CR45] Rittig-Rasmussen B, Kasch H, Fuglsang-Frederiksen A, Svensson P, Jensen TS (2014). Effect of training on corticomotor excitability in clinical neck pain. European Journal of Pain.

[CR46] Rittig-Rasmussen B, Kasch H, Fuglsang-Frederiksen A, Svensson P, Jensen TS (2014). The role of neuroplasticity in experimental neck pain: A study of potential mechanisms impeding clinical outcomes of training. Manual Therapy.

[CR47] Romaniello A, Cruccu G, McMillan AS, Arendt-Nielsen L, Svensson P (2000). Effect of experimental pain from trigeminal muscle and skin on motor cortex excitability in humans. Brain Research.

[CR48] Rosso, C., & Lamy, J.-C. (2018). Does Resting Motor Threshold Predict Motor Hand Recovery After Stroke? *Frontiers in Neurology*, 9. 10.3389/fneur.2018.01020 10.3389/fneur.2018.01020 PMC6281982 30555404

[CR49] Rothwell, J. (2018). Transcranial brain stimulation: Past and future. *Brain and Neuroscience Advances*, 2, 239821281881807. 10.1177/2398212818818070 10.1177/2398212818818070 PMC7058222 32166172

[CR50] Schwenkreis P, Voigt M, Hasenbring M, Tegenthoff M, Vorgerd M, Kley RA (2011). Central Mechanisms during Fatiguing Muscle Exercise in Muscular Dystrophy and Fibromyalgia Syndrome: A Study with Transcranial Magnetic Stimulation. Muscle & Nerve.

[CR51] Stang A (2010). Critical evaluation of the Newcastle-Ottawa scale for the assessment of the quality of nonrandomized studies in meta-analyses. European Journal of Epidemiology.

[CR52] Stefan K, Wycislo M, Classen J (2004). Modulation of associative human motor cortical plasticity by attention. Journal of Neurophysiology.

[CR53] Svensson P, Romaniello A, Arendt-Nielsen L, Sessle BJ (2003). Plasticity in corticomotor control of the human tongue musculature induced by tongue-task training. Experimental Brain Research.

[CR54] Tsao H, Galea MP, Hodges PW (2010). Driving plasticity in the motor cortex in recurrent low back pain. European Journal of Pain.

[CR55] Vallence AM, Smith A, Tabor A, Rolan PE, Ridding MC (2013). Chronic tension-type headache is associated with impaired motor learning. Cephalalgia.

[CR56] Volz, M. S., Mendonca, M., Pinheiro, F. S., Cui, H., Santana, M., Fregni, F., & Zhan, W. (2012). Dissociation of motor task-induced cortical excitability and pain perception changes in healthy volunteers. *PLoS ONE,**7*(3), e34273. 10.1371/journal.pone.0034273 10.1371/journal.pone.0034273 PMC3314609 22470548

[CR57] Wang R, Ke S, Zhang Q, Zhou K, Li P, Yang J (2020). Functional and structural neuroplasticity associated with second language proficiency: An MRI study of Chinese-English bilinguals. Journal of Neurolinguistics.

[CR58] Wulf G, Shea C, Lewthwaite R (2010). Motor skill learning and performance: A review of influential factors. Medical Education.

