# Supplementary material for: Pain’s Adverse Impact on Training-Induced Performance and Neuroplasticity: A Systematic Review
Source: Brain Imaging Behav. 2022 Mar 18;16(5):2281–306. doi: 10.1007/s11682-021-00621-6 (PMC9581826; doi:10.1007/s11682-021-00621-6)
Supplement: Supplementary file 1 — Supplementary file1 (PDF 614 kb) [file 11682_2021_621_MOESM1_ESM.pdf]

**Checklist for assessing methodological quality of TMS studies**	**Baarbe 2018**		**Boudreau 2007**		**Dancey 2019**	
**Were the following participant factors:**	**Reported**	**Controlled**	**Reported**	**Controlled**	**Reported**	**Controlled**
Age of subjects	Yes	No	Yes	No	Yes	No
Gender of subjects	Yes	N/A	Yes	N/A	Yes	N/A
Handedness of subjects	Yes	Yes	No	No	Yes	No
Subjects prescribed medication	No	No	No	No	No	No
Use of CSN active drugs (e.g. anti-convulsant)	No	No	No	No	No	No
Presence of neurological/psychiatric disorders when studying healthy subjects	Yes	Yes	Yes	Yes	Yes	Yes
Any medical conditions	Yes	Yes	Yes	Yes	Yes	Yes
History of specific repetitive motor activity	No	No	No	No	No	No
**Were the following methodological factors:**	**Reported**	**Controlled**	**Reported**	**Controlled**	**Reported**	**Controlled**
Position and contact of EMG electrodes	Yes	Yes	Yes	Yes	Yes	Yes
Amount of relexation/contraction of target muscles	Yes	Yes	Yes	No	Yes	Yes
Prior motor activity of the muscle to be tested	Yes	No	Yes	No	Yes	Yes
Level of relaxation of muscles other than those being tested	N/A	No	N/A	Yes	N/A	Yes
Coil type (size and geometry)	Yes	Yes	Yes	Yes	Yes	Yes
Coil orientation	Yes	Yes	Yes	Yes	Yes	Yes
Direction of induced current in the brain	No	No	Yes	Yes	Yes	Yes
Coil location and stability	No	No	Yes	Yes	Yes	Yes
Type of stimulation used (e.g. brand)	Yes	Yes	Yes	Yes	Yes	Yes
Stimulation intensity	Yes	N/A	Yes	Yes	Yes	Yes
Pulse shape (monophasic or biphasic)	No	No	No	No	No	No
Determination of optimal hotspot	No	No	Yes	Yes	Yes	Yes
The time between MEP trials	No	No	Yes	Yes	Yes	Yes
Times between days of testing	N/A	N/A	Yes	Yes	N/A	N/A
Subject attention (level of arousel) during testing	No	No	No	No	No	No
Method for determining threshold (active/resting)	Yes	Yes	Yes	Yes	Yes	Yes
Number of MEP measures made	Yes	Yes	Yes	Yes	Yes	Yes
Paired puls only: Intensity of test puls	N/A	N/A	N/A	N/A	N/A	N/A
Paired puls only: Intensity of conditioning puls	N/A	N/A	N/A	N/A	N/A	N/A
Paired puls only: Inter-stimulus interval	N/A	N/A	N/A	N/A	N/A	N/A
**Were the following analytical factors:**	**Reported**	**Controlled**	**Reported**	**Controlled**	**Reported**	**Controlled**
Method for determining MEP size during analysis	Yes	Yes	Yes	Yes	Yes	Yes
Size of unconditioned MEP	N/A	N/A	N/A	N/A	N/A	N/A

De Martino 2018a		De Martino 2018b		Hoeger Bement 2014		Ingham 2011		Masse Alaire 2014		Masse Alaire 2016	
**Reported**	**Controlled**	**Reported**	**Controlled**	**Reported**	**Controlled**	**Reported**	**Controlled**	**Reported**	**Controlled**	**Reported**	**Controlled**
Yes	Yes	Yes	Yes	Yes	Yes	Yes	Yes	Yes	Yes	Yes	Yes
Yes	N/A	Yes	N/A	Yes	N/A	Yes	N/A	Yes	N/A	Yes	N/A
Yes	Yes	Yes	Yes	No	No	No	No	Yes	Yes	No	No
No	No	No	No	No	No	No	No	No	No	No	No
No	No	No	No	No	No	No	No	Yes	Yes	Yes	Yes
Yes	Yes	Yes	Yes	Yes	No	No	No	Yes	Yes	Yes	Yes
Yes	Yes	Yes	Yes	No	No	Yes	Yes	Yes	Yes	Yes	Yes
No	No	No	No	No	No	No	No	No	No	No	No
**Reported**	**Controlled**	**Reported**	**Controlled**	**Reported**	**Controlled**	**Reported**	**Controlled**	**Reported**	**Controlled**	**Reported**	**Controlled**
Yes	Yes	Yes	Yes	Yes	Yes	Yes	Yes	Yes	Yes	Yes	Yes
Yes	No	No	Yes	Yes	No	Yes	Yes	Yes	Yes	Yes	Yes
No	No	No	Yes	No	No	Yes	Yes	Yes	Yes	Yes	Yes
N/A	No	N/A	No	N/A	No	N/A	Yes	N/A	No	N/A	No
Yes	Yes	Yes	Yes	Yes	Yes	Yes	Yes	Yes	Yes	Yes	Yes
No	No	Yes	Yes	No	No	Yes	Yes	No	Yes	No	Yes
No	No	Yes	Yes	No	No	Yes	Yes	No	No	No	No
Yes	No	Yes	No	Yes	No	Yes	Yes	Yes	Yes	Yes	Yes
Yes	Yes	Yes	Yes	Yes	Yes	Yes	Yes	Yes	Yes	Yes	Yes
Yes	Yes	Yes	Yes	Yes	Yes	Yes	Yes	Yes	Yes	Yes	Yes
No	No	No	No	No	No	Yes	Yes	Yes	Yes	Yes	Yes
Yes	Yes	Yes	Yes	No	No	Yes	Yes	Yes	Yes	Yes	Yes
Yes	Yes	Yes	Yes	Yes	Yes	No	No	No	No	No	No
Yes	Yes	Yes	Yes	N/A	N/A	Yes	Yes	Yes	Yes	Yes	Yes
No	No	No	No	No	No	No	No	No	No	No	No
Yes	Yes	Yes	Yes	Yes	Yes	Yes	Yes	Yes	Yes	Yes	Yes
Yes	Yes	Yes	Yes	Yes	Yes	Yes	Yes	Yes	Yes	Yes	Yes
N/A	N/A	N/A	N/A	N/A	N/A	N/A	N/A	Yes	Yes	Yes	Yes
N/A	N/A	N/A	N/A	N/A	N/A	N/A	N/A	Yes	Yes	Yes	Yes
N/A	N/A	N/A	N/A	N/A	N/A	N/A	N/A	Yes	Yes	Yes	Yes
**Reported**	**Controlled**	**Reported**	**Controlled**	**Reported**	**Controlled**	**Reported**	**Controlled**	**Reported**	**Controlled**	**Reported**	**Controlled**
Yes	Yes	Yes	Yes	Yes	Yes	Yes	Yes	Yes	Yes	Yes	Yes
N/A	N/A	N/A	N/A	N/A	N/A	N/A	N/A	Yes	Yes	Yes	Yes

Mavromatis 2016		Mendonca 2016		Parker 2017		Rittig-Rasmussen 2014a		Rittig-Rasmussen 2014b		Schwenkreis 2011	
**Reported**	**Controlled**	**Reported**	**Controlled**	**Reported**	**Controlled**	**Reported**	**Controlled**	**Reported**	**Controlled**	**Reported**	**Controlled**
Yes	Yes	Yes	Yes	Yes	Yes	Yes	Yes	Yes	Yes	Yes	Yes
Yes	N/A	Yes	N/A	Yes	N/A	Yes	N/A	Yes	N/A	Yes	N/A
Yes	Yes	Yes	No	Yes	No	No	No	No	No	Yes	Yes
No	No	No	No	No	No	Yes	Yes	No	No	No	No
No	No	No	No	Yes	Yes	No	No	No	No	Yes	Yes
Yes	Yes	N/A	N/A	Yes	Yes	Yes	Yes	Yes	Yes	Yes	Yes
Yes	Yes	Yes	Yes	Yes	Yes	Yes	Yes	Yes	Yes	Yes	Yes
No	No	No	No	No	No	No	No	No	No	No	No
**Reported**	**Controlled**	**Reported**	**Controlled**	**Reported**	**Controlled**	**Reported**	**Controlled**	**Reported**	**Controlled**	**Reported**	**Controlled**
Yes	Yes	No	No	Yes	Yes	Yes	Yes	Yes	Yes	Yes	Yes
Yes	No	No	No	Yes	Yes	Yes	No	Yes	No	Yes	Yes
No	No	No	No	Yes	Yes	Yes	No	Yes	No	Yes	Yes
N/A	No	N/A	No	N/A	Yes	N/A	No	N/A	No	N/A	Yes
Yes	Yes	Yes	Yes	Yes	Yes	Yes	Yes	Yes	Yes	Yes	Yes
Yes	Yes	No	No	Yes	Yes	Yes	Yes	Yes	Yes	Yes	Yes
Yes	Yes	No	No	Yes	Yes	Yes	Yes	Yes	Yes	No	No
Yes	Yes	No	No	Yes	Yes	Yes	Yes	Yes	Yes	No	No
Yes	Yes	Yes	Yes	Yes	Yes	Yes	Yes	Yes	Yes	Yes	Yes
Yes	Yes	Yes	Yes	Yes	Yes	Yes	Yes	Yes	Yes	No	No
Yes	Yes	No	No	Yes	Yes	No	No	No	No	No	No
Yes	Yes	Yes	Yes	Yes	Yes	Yes	Yes	Yes	Yes	Yes	Yes
No	No	No	No	Yes	Yes	Yes	Yes	Yes	Yes	No	No
N/A	N/A	Yes	Yes	Yes	No	Yes	Yes	Yes	Yes	N/A	N/A
No	No	No	No	Yes	Yes	No	No	No	No	No	No
Yes	Yes	Yes	Yes	Yes	Yes	Yes	Yes	Yes	Yes	Yes	Yes
Yes	Yes	Yes	Yes	Yes	Yes	Yes	Yes	Yes	Yes	Yes	Yes
Yes	Yes	Yes	Yes	Yes	Yes	N/A	N/A	N/A	N/A	No	No
Yes	Yes	Yes	Yes	Yes	Yes	N/A	N/A	N/A	N/A	Yes	Yes
No	No	Yes	Yes	Yes	Yes	N/A	N/A	N/A	N/A	Yes	Yes
**Reported**	**Controlled**	**Reported**	**Controlled**	**Reported**	**Controlled**	**Reported**	**Controlled**	**Reported**	**Controlled**	**Reported**	**Controlled**
Yes	Yes	Yes	Yes	Yes	Yes	Yes	Yes	Yes	Yes	Yes	Yes
No	Yes	No	No	No	No	N/A	N/A	N/A	N/A	No	No

Tsao 2010		Vallence 2013	
**Reported**	**Controlled**	**Reported**	**Controlled**
Yes	Yes	Yes	Yes
Yes	N/A	Yes	N/A
Yes	Yes	Yes	Yes
No	No	No	No
No	No	Yes	Yes
N/A	N/A	Yes	Yes
Yes	Yes	Yes	Yes
No	No	No	No
**Reported**	**Controlled**	**Reported**	**Controlled**
Yes	Yes	Yes	Yes
Yes	Yes	Yes	Yes
Yes	Yes	No	No
N/A	No	N/A	N/A
Yes	Yes	Yes	Yes
Yes	Yes	Yes	Yes
Yes	Yes	Yes	Yes
Yes	Yes	Yes	Yes
Yes	Yes	Yes	Yes
Yes	Yes	No	No
No	No	No	No
No	No	Yes	Yes
Yes	Yes	Yes	Yes
Yes	Yes	N/A	N/A
No	No	No	No
No	No	Yes	Yes
Yes	Yes	Yes	Yes
N/A	N/A	N/A	N/A
N/A	N/A	N/A	N/A
N/A	N/A	N/A	N/A
**Reported**	**Controlled**	**Reported**	**Controlled**
Yes	Yes	Yes	Yes
N/A	N/A	N/A	N/A
